# Complexes of Hydrogen Peroxide, the Simplest Chiral Molecule, with L- and D-Serine Enantiomers and Their Clusters: MP2 and DFT Calculations†

**DOI:** 10.3390/molecules29163955

**Published:** 2024-08-21

**Authors:** Yurii A. Borisov, Sergey S. Kiselev, Mikhail I. Budnik, Lubov V. Snegur

**Affiliations:** 1 Federal State Budgetary Scientific Institution, A.N. Nesmeyanov Institute of Organoelement Compounds, Russian Academy of Science, 28 Vavilov St., 119991 Moscow, Russia; yuaborisov@mail.ru (Y.A.B.); kiss@ineos.ac.ru (S.S.K.); 2 Federal State Budgetary Scientific Institution, N.N. Semenov Federal Research Center of Chemical Physics, Russian Academy of Science, 4 Kosygin St., 119991 Moscow, Russia; ziraf@mail.ru

**Keywords:** hydrogen peroxide, complexes, L- and D-serine, MP2 and DFT calculations, CD spectra

## Abstract

The interaction between natural amino acids and hydrogen peroxide is of paramount importance due to the widespread use of hydrogen peroxide in biological and environmentally significant processes. Given that both amino acids and hydrogen peroxide occur in nature in two enantiomeric forms, it is crucial to investigate the formation of complexes between them, considering the role of molecular chirality. In this work, we report a theoretical study on the hydrogen peroxide enantiomers and their interactions with L- and S-serine and their clusters. We aimed to evaluate the non-covalent interactions between each hydrogen peroxide enantiomer and the L- and D-enantiomers of the non-essential amino acid serine and their clusters. First, the potential energy surfaces (PES) of transitions between enantiomers of the simplest chiral molecule, hydrogen peroxide, in the gas phase and in aqueous solution were studied using the Møller–Plesset theory method MP2/aug-cc-pVDZ. The activation energies of such transitions were calculated. The interactions of both hydrogen peroxide enantiomers (P and M) with L- and D-serine enantiomers were analyzed by density functional theory (DFT) with ωb97xd/6-311+G**, B3Lyp/6-311+G**, B3P86/6-311+G**, and M06/6-311+G** functionals. We found that both enantiomers of hydrogen peroxide bind more strongly to L-serine and its clusters than to D-serine, especially highlighting that the L form is the predominant natural form of this and other chiral amino acids. The optimized geometric parameters, interaction energies, and HOMO-LUMO energies for various complexes were estimated. Furthermore, circular dichroism (CD) spectra, which are optical chirality characteristics, were simulated for all the complexes under study.

## 1. Introduction

**Figure 1:**
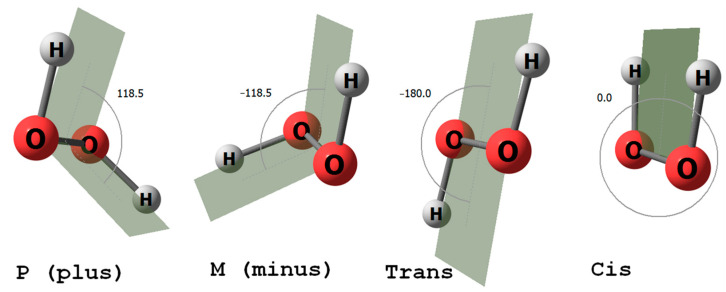
H_2_O_2_ molecule: P and M axial chiral forms (on the (**left**)) and *trans*-*cis* forms (on the (**right**)).

All living things on Earth are homochiral. Is this a trivial or non-trivial conclusion? All schoolchildren know that the biology of living things uses only the L-enantiomers of chiral amino acids and the D-enantiomer of ribose, the sugar fragment of ribonucleic acids. Less well known is that hydrogen peroxide, the smallest and simplest molecule, exists as a pair of enantiomers (Figure 1). H_2_O_2_ is a non-planar molecule with a twisted C2 symmetry, first demonstrated by Giger in 1950 using infrared spectroscopy [1]. In 2011, hydrogen peroxide was detected in the interstellar medium with an abundance of HOOH relative to H_2_ of about 1 × 10^−10^ [2]. Ball and Brindley proposed that the enantiospecific interaction between hydrogen peroxide, ribose, and amino acids results in enantioselectivity, leading to homochirality [3]. The findings of this study [3], which detailed the role of hydrogen peroxide in driving vibrational motion in the RNA world, prompted us to investigate further, focusing specifically on the interactions of hydrogen peroxide with amino acid enantiomers. Previous studies have developed and examined schemes for controlling chirality in macroscopic volumes using short near-infrared laser pulses, with hydrogen peroxide molecules as an example [4]. These studies considered possible experimental conditions and various approaches to chirality detection.

Theoretical studies with the chirality of hydrogen peroxide, on discriminating between different chiral forms of organic molecules or complexes H-bonded with hydrogen peroxide, have been performed in several papers [5,6,7,8]. The so-called chiral discriminating interactions represent the interaction energy Δ*E* between two molecules, both of which can exist in chiral R- and S-enantiomers, depending on their relative handedness. Dong et al. investigated four complexes using DFT and MP2 theory [5]. The studied complexes were formed between two isolated chiral hydrogen peroxide molecules (M and P) and two chiral R- and S-lactic acid molecules. The discrimination between these forms was identified [5]. Du and Zhou studied the discrimination of different chiral forms of 1:1 hydrogen peroxide complexes with methyl hydroperoxide using density functional theory (DFT) and Møller–Plesset type 2 (MP2) methods on different basis sets ranging from 6-31G (d, p) to 6-31G (2d, 2p) [6]. Yin and co-authors analyzed three pairs of chiral enantiomers [7]. A theoretical study was conducted on the chiral discrimination of various chiral formates of hydrogen-bonded butan-2-ol complexes with hydrogen peroxide [7]. Zang et al. investigated the effect of chirality on intermolecular interactions between two chiral molecules connected by hydrogen bonds [8]. The authors employed a second-order Møller–Plesset perturbation theory (MP2) method with the 6-311G (d, p) basis set. Four diastereomeric complexes were formed via the hydrogen bond between chiral (*S*)-oxirane 2-methylol and chiral HOOH molecules (P and M). The CD spectra of the compounds and complexes were calculated [8].

CD spectroscopy is a widely used method for analyzing mixtures of optical isomers of biologically active compounds and for quality control of drugs. It is also commonly used to determine the amount of protein and monitor its secondary structure in solutions, such as denaturation changes [9].

This study aims to analyze the interactions between both enantiomers of hydrogen peroxide (P and M) and L- and D-serine enantiomers as well as clusters of L-serine using the DFT computational methods. We also report the results of quantum chemical calculations of the potential energy surfaces (PES) for the transitions between two hydrogen peroxide enantiomers in the gas phase and in aqueous solution. In the study, the calculated total energies, dipole moments, and HOMO-LUMO energies for L-serine and its dimer and tetramer clusters were analyzed. In addition, CD spectra, as a characteristic of optical chirality, were simulated for all studied complexes in both gas and aqueous phases. The use of predicted calculated CD spectra allows for the reliable assignment of the experimental CD spectra of the complexes under study.

## 2. Results and Discussion

The main goal of this work was to calculate the energy interactions between enantiomers of hydrogen peroxide and the amino acid serine enantiomers in order to estimate priorities. We believe that the patterns identified will allow to better and more deeply understand the role of hydrogen peroxide in the origin of life on Earth.

This section first considers the trans-cis energy transitions observed in the hydrogen peroxide molecule. The following are the calculated total energies, dipole moments, and HOMO-LUMO energies for L-serine and its clusters. The primary focus of Section 3 is the calculation of the energy characteristics and structures of hydrogen peroxide complexes with enantiomers of L-serine and D-serine as well as their dimeric and tertamer clusters.

### 2.1. M-P Transition Energies of Hydrogen Peroxide in the Gas Phase and in Aqueous Solution

The H_2_O_2_ molecule in the gas phase is known to have two axial chiral forms, P and M [10]. In this paper, we consider the dihedral angle of HOOH to be greater than zero for the P (plus) form and less than zero for the M (minus) form (Figure 1).

**Figure 2:**
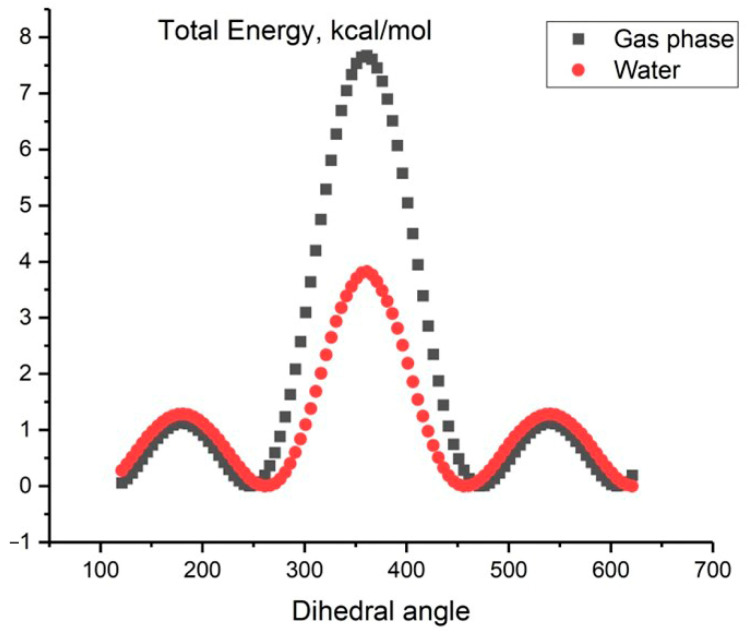
The dependence of the total energy *E* (MP2/aug-cc-pVDZ) on the HOOH dihedral angle for H_2_O_2_ in the gas phase (black line) and in aqueous solution (red line) using the continuum solvent SMD model (calculated data).

Figure 2 displays the relationship between the total energy *E* (MP2/aug-cc-pVDZ) and the dihedral angle of the HOOH. There are two barriers to transitioning between forms: a high barrier, referred to as *cis* form in the literature, and a low barrier, known as *trans* form. Our calculations show that the activation energy for the transition state, TS*cis*, is 7.21 kcal/mol and for TS*trans* is 1.26 kcal/mol. These values are in accordance with the experimental spectral data of far-infrared absorption spectra of hydrogen peroxide, which report values of 2460 cm^−1^ (or 7.03 kcal/mol) and 386 cm^−1^ (or 1.10 kcal/mol), respectively, for *cis* and *trans* potential barrier heights [11].

Similar calculations were conducted by our research group to investigate the dependence of the total energy, *E* (MP2/aug-cc-pVDZ), on the HOOH dihedral angle for an aqueous solution using the continuum SMD solvent model. The results are presented in Figure 2.

In this case, the activation energy for *E* (TS*cis*) is 4.73 kcal/mol and for *E* (TS*trans*) is 2.20 kcal/mol. The presence of an aqueous solvent increases the low barrier by 0.94 kcal and decreases the high barrier by 2.48 kcal. This results in a decrease in the energy gap in an aqueous peroxide solution compared to the gas phase (Figure 2, red line).

The Supplementary Materials display a plot of the total energy *E* (MP2/aug-cc-pVDZ) as a function of the HOOH dihedral angle for the (H_2_O_2_)_2_ dimer in aqueous solution for the continuum solvent SMD model. The *cis*-orientation of the dimer has an activation energy of 4.77 kcal/mol, while the *trans*-orientation has an activation energy of 2.28 kcal/mol. As can be observed, the underlying meanings are essentially identical. The (H_2_O_2_)*_n_* (for *n* = 2–4) clusters were previously studied ab initio in reference [12].

**Figure 3:**
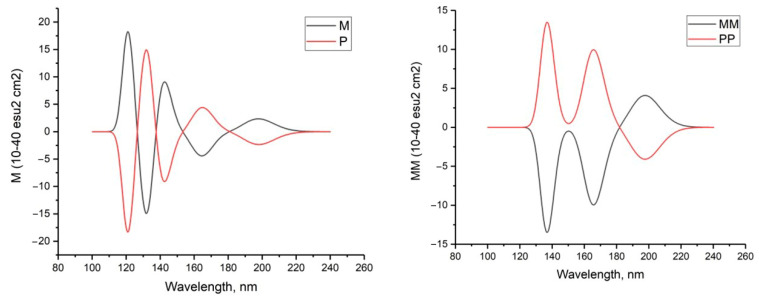
Calculated CD spectra of hydrogen peroxide in M and P forms (**left**) and its dimers (**right**); MP2/6-311+G**.

Circular dichroism in the UV range can be used to characterize hydrogen peroxide due to its optical activity. The CD spectra of the investigated complexes unambiguously demonstrate the distinctions between enantiomers exhibiting positive or negative maximums of the Cotton effect. This is particularly evident in Figure 3. Figure 3 shows the calculated CD spectra for hydrogen peroxide molecules in both M (negative dihedral angle HOOH) and P (positive dihedral angle HOOH) configurations as well as dimers (M-M and P-P). The calculated CD spectra of cyclic tetramers (M-M-M-M, M-P-M-P, and P-P-P-P) can be seen in the Supplementary Materials. As anticipated, the CD spectra of M- and P-peroxide as well as the dimers M-M and P-P exhibit a completely symmetrical appearance.

The structures of the dimers, cyclic trimers, and cyclic tetramers are provided in the Supplementary Materials.

### 2.2. Clusters of Serine Molecules

Prior to examining the interactions between hydrogen peroxide and serine’s enantiomers, it is first necessary to analyze the serine molecule and clusters of serine. This section presents the results of the L-serine cluster calculations. The complexes’ structure was determined, and the electronic characteristics and energetics of the transitions between the different clusters were established. Table 1 presents the results of calculations, including the energies of the highest occupied molecular orbitals (HOMO) and the lowest unoccupied molecular orbitals (LUMO) for L-serine and clusters of L-serine, i.e., (L-Ser)*_n_*, *n* = 2–16, using the DFT method with the ωb97xd/6-311+G* basis set.

The Supplementary Materials include two figures depicting the structures of L-serine clusters and Cartesian coordinates of the atoms. Figure S2 displays a cluster of four L-serine molecules, while Figure S3 shows a cluster of eight L-serine molecules.

The relative stability of L-serine clusters can be characterized by the following value:

Δ*E* = [*E*(*n*-Ser) − *nE*(Ser)]/*n* (1)

where *E*(*n*-Ser) is the total energy of a cluster containing *n* L-serine molecules, and *E*(Ser) is the total energy of L-serine.

**Figure 4:**
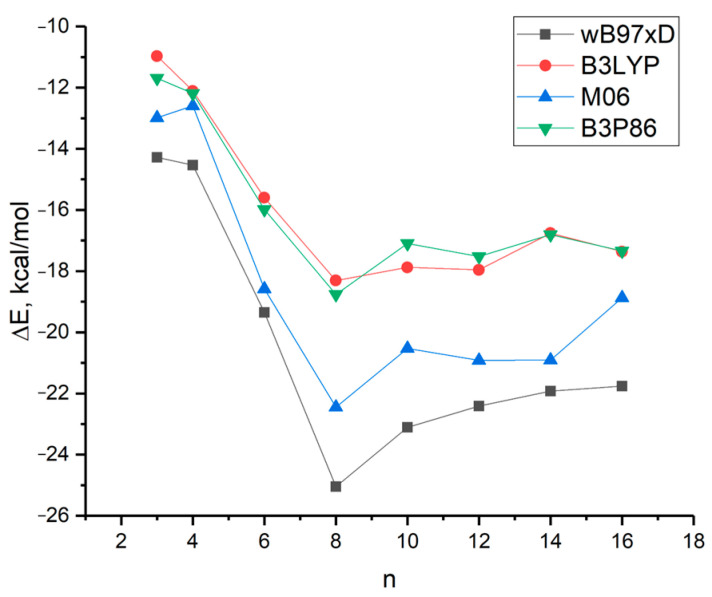
Dependence of the value of Δ*E* = [*E*(*n*-Ser) − *nE*(Ser)]/*n* in kcal per mole on the cluster size of *n* L-serine molecules.

Figure 4 illustrates the relationship between the energy Δ*E* in kcal/mol and the size of *n* L-serine clusters, as obtained by four different DFT methods using the 6-31G* basis set.

The Supplementary Materials of this paper present the results of calculations of clusters of L-serine, i.e., *n*-Ser-L, from *n* = 1 to *n* = 16 using DFT B3LYP, M06, and B3P86 methods.

The curves obtained by four different DFT methods exhibit a similar nature, characterized by a sharp increase in cluster stability from *n* = 3 to *n* = 8. The latter represents the most stable cluster according to the calculations. Beyond *n* = 10, cluster stability either decreases monotonically (curves black and blue) or remains relatively constant (curves red and green).

The calculated data are in good agreement with the predominant percentage of serine clusters, with *n* = 8 observed in the mass spectrum of L-serine during electrospray ionization [13,14]. Serine octamer clusters (Ser8) have received considerable research attention due to their homochirality and their potential role in the origin of life [15,16].

### 2.3. Interaction of M- and P-Hydrogen Peroxide with L- and D-Serine Enantiomers

**Figure 5:**
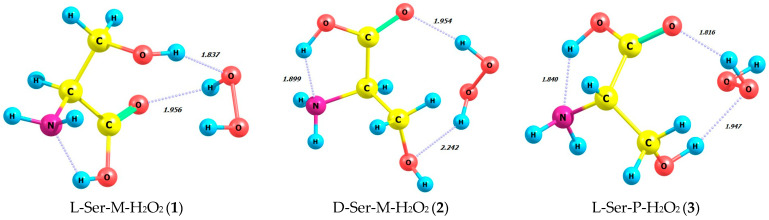

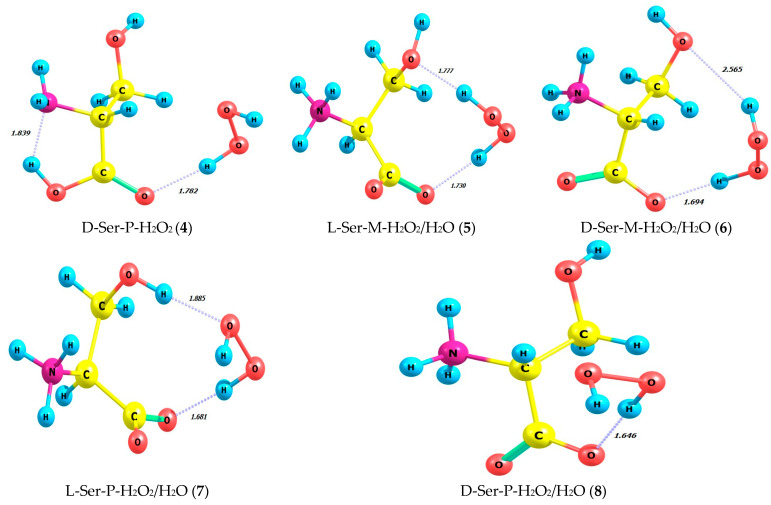
Structures of hydrogen peroxide complexes in M and P forms with L- and D-enantiomers of serine in gas phase and in aqueous solution; MP2/aug-cc-pVDZ calculation method.

Table 2 and Figure 5 summarize energy characteristics and the structures of hydrogen peroxide complexes in M and P forms with L- and D-serine enantiomers in the gas phase (**1**–**4**) and in aqueous solution (**5**–**8**). Complexes **1**–**4** correspond to structures **5**–**8**, taking into account the impact of the aqueous solvent in the SMD model. The zwitterionic form of serine is evident in the aqueous solution of complexes **5**–**8**. Table S12 provides supplementary data on bond lengths including hydrogen ones and bond angles for the complexes **1**–**8** depicted in Figure 5. Interestingly, the total energies and the energies of frontier orbitals, i.e., HOMO-LUMO, calculated for the complexes **1**–**8** have quite close values.

It is important to consider the non-covalent interactions in these complexes more thoroughly. When comparing the complexes L-Ser-M-H_2_O_2_ (**1**) and D-Ser-M-H_2_O_2_ (**2**) (Figure 5), it is evident that L-serine is linked to only one part of the M-peroxide molecule through two hydrogen bonds. These H-bonds are formed by both hydrogen of the hydroxyl group of L-serine and its carboxylic oxygen. In the complex D-Ser-M-H_2_O_2_ (**2**) (Figure 5) with the same M-peroxide, two intermolecular hydrogen bonds are observed. However, both hydrogen from the peroxide side and two L-Ser oxygens, namely hydroxyl and carboxyl, are involved in their formation. According to Table 2, the most energetically favorable complex is L-Ser-M-H_2_O_2_ (**1**), with energy of −549.229 a.u. compared to **2**.

When comparing another pair of complexes, namely L-Ser-P-H_2_O_2_ (**3**) and D-Ser-P-H_2_O_2_ (**4**) (Table 2), it is noteworthy that L-serine as well as in the complex with M-peroxide (**1**) forms two hydrogen bonds with only one part of the P-peroxide molecule. Both of these hydrogen bonds, similar to **1**, are formed by serine’s hydroxyl hydrogen and its carboxylic oxygen. Therefore, the L-Ser-P-H_2_O_2_ (**3**) complex is the most energetically favorable, with *E* = −549.232 a.u., out of the four considered: **1**, **2**, **3**, and **4**. In complex **4**, D-Ser-P-H_2_O_2_ forms only one hydrogen bond, which is the shortest at 1.782 Å, between the carboxylic oxygen and P-peroxide (Figure 5).

Calculations that take into account the solvent (H_2_O, continuum model) reveal differences from the results obtained for the gas phase (Table 2; complexes **5**, **6**, **7**, and **8**). In the aqueous solution of the L-Ser-M-H_2_O_2_ complex (**5**), both hydrogen atoms of peroxide and two oxygen atoms of L-Ser (β-hydroxyl and carboxylic) form H-bonds on the M-peroxide side, whereas in the gas phase, only part of the peroxide molecule is involved in the same complex. In the D-Ser-M-H_2_O_2_ complex (**6**) for aqueous solution, D-Ser is bound by two H-bonds, formed analogously to complex **5** between the carboxylic and hydroxyl oxygens of serine’s molecule and hydrogens of M-peroxide, and one of the H-bonds is the longest, equal to 2.565 Å, out of all eight complexes. Finally, the remaining pair of L-Ser and D-Ser complexes with P-peroxide (Table 2; complexes **7** and **8**) forms two and one hydrogen bonds, respectively, in aqueous solution. Additionally, in complex **7**, the hydrogen of the β-hydroxyl of L-Ser forms an H-bond.

Note that in Figure 5, for complexes **5**, **6**, **7**, and **8**, serine is shown as a zwitterion, meaning that the amino group is protonated (−NH_3_^+^), and the carboxyl group is deprotonated (−COO^−^).

Using the data presented in Table 2, we computed the interaction energies in both the gas phase and in aqueous solution using the SMD model, taking into account the impact of the aqueous solvent (see Table 3).

Based on the data, it can be concluded that the L-serine stereoisomer has a stronger binding affinity to the hydrogen peroxide molecule (both P and M form) in both gas phase and aqueous solution (as shown in Table 3, lines 1, 3, 5, and 7).

**Figure 6:**
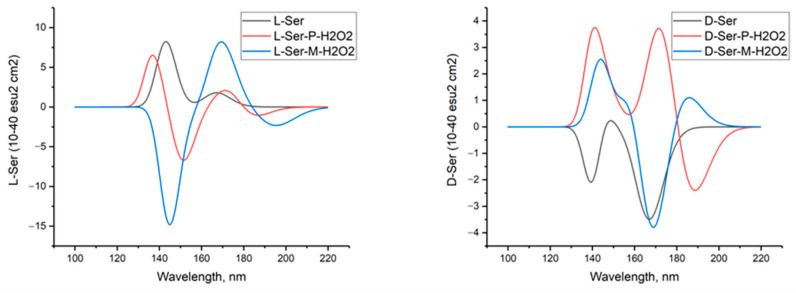
Circular dichroism spectra of L-serine, D-serine, and their complexes with H_2_O_2_ in aqueous solution; MP2/6-311+G**.

Figure 6 shows the calculated circular dichroism spectra of L-serine, D-serine, and their complexes with H_2_O_2_ in aqueous solution.

### 2.4. Complexes of Two Serine Molecules and Their Complexes with Two Hydrogen Peroxide Molecules, DFT Calculation Method ωb97xd/6-311+G**

**Figure 7:**
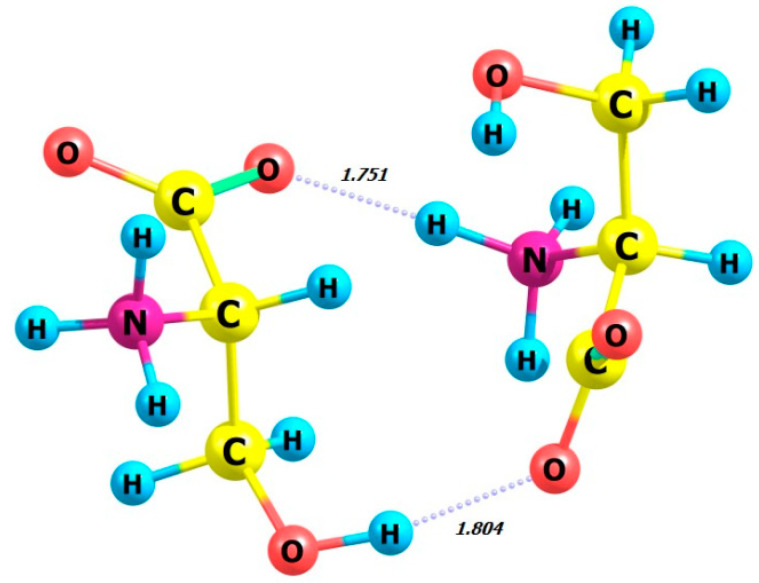
Structure of L-serine dimer in aqueous solution.

**Figure 8:**
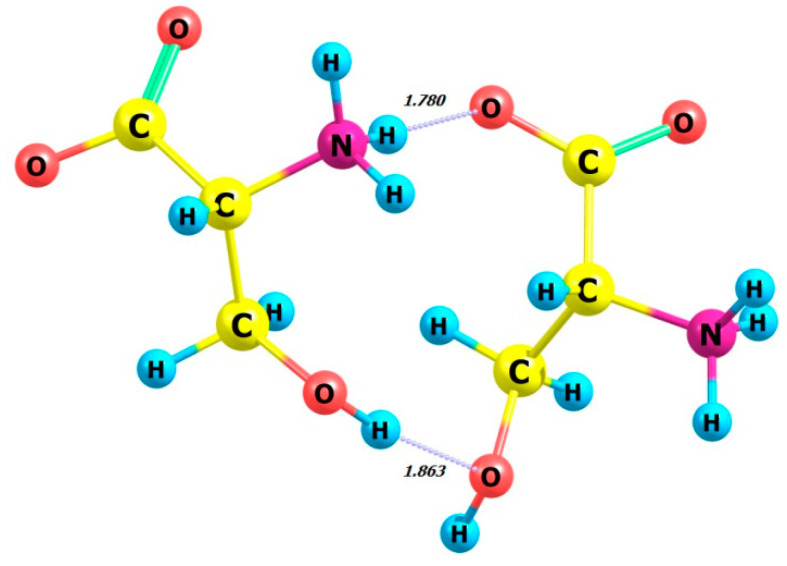
Structure of D-serine dimer in aqueous solution.

**Figure 9:**
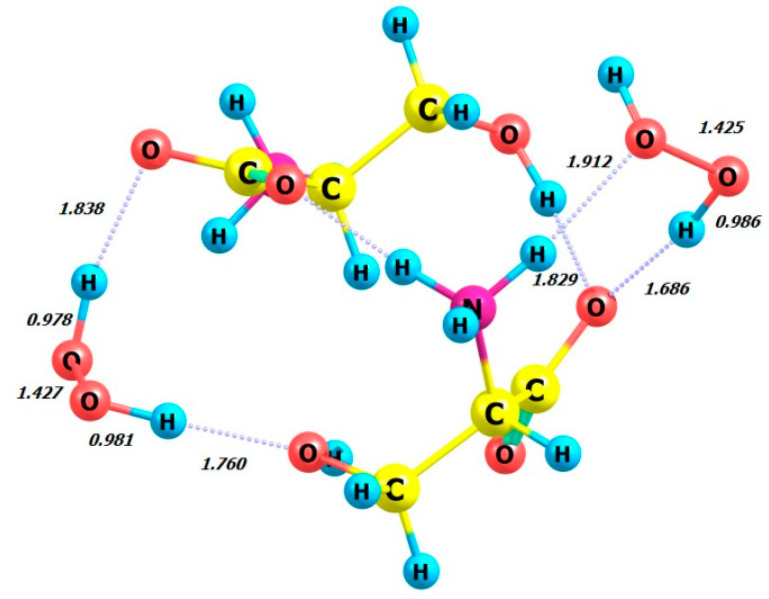
Structure of L-serine dimer with two hydrogen peroxide (MM) molecules in aqueous solution.

**Figure 10:**
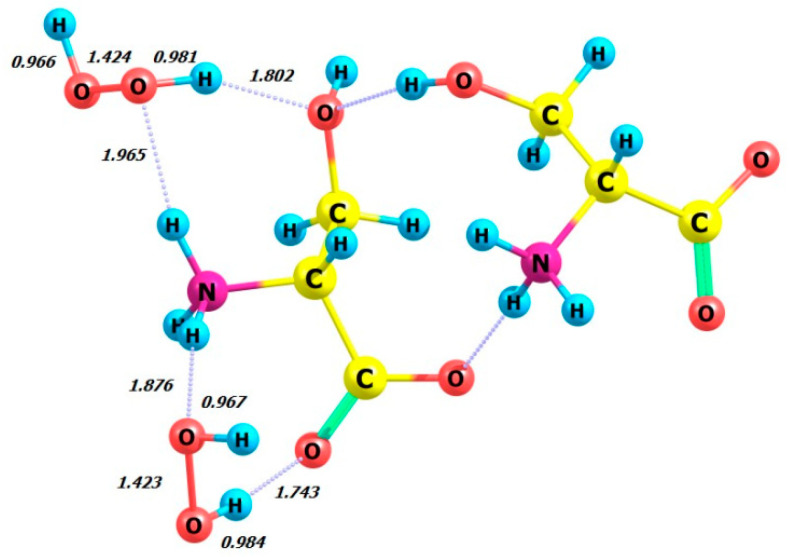
Structure of D-serine dimer with two hydrogen peroxide (MM) molecules in aqueous solution.

Next, we examine the dimers of L-serine and D-serine as well as their complexes with hydrogen peroxide in M form. Figure 7, Figure 8, Figure 9 and Figure 10 display the structures of L- and D-serine dimers and their complexes with two hydrogen peroxide molecules.

Table 4 presents the calculated dihedral angles in hydrogen peroxide and its complexes as well as the total energies and HOMO and LUMO energies of serine dimers and their complexes with H_2_O_2_. The energy changes Δ*E* (see the sixth column) for the following interactions were calculated based on the data presented in Table 4.

Regarding the energetics of the presented processes, it is noteworthy that obtaining L-serine as a dimer and its participation as a complex with M-peroxide are the most favorable processes compared to the D-serine-based analogues.

The energy gain from forming a complex with the L-serine dimer is significantly higher than with the L-serine monomer (compare *E* = −18.14 kcal/mol, Table 4, and −10.91 kcal/mol, Table 3, respectively).

**Figure 11:**
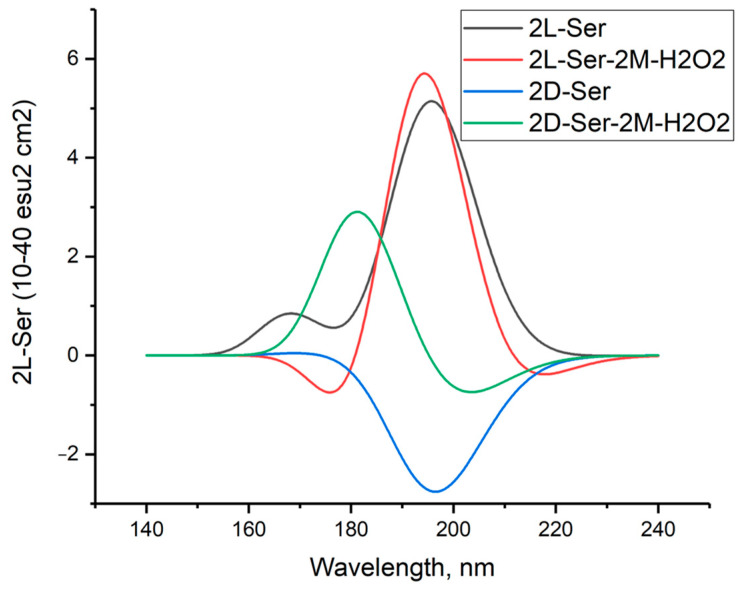
Circular dichroism spectra of L- and D-serine dimers and their complexes with two molecules of hydrogen peroxide in M form in aqueous solution; DFT calculation method ωb97xd/6-311+G**.

Figure 11 shows the simulated circular dichroism spectra of L- and D-serine dimers as well as their complexes with two hydrogen peroxide molecules in the M form. The Supplementary Materials provide a comparison of the CD spectra of these compounds obtained using three other DFT methods.

The comparison between the calculated CD spectrum of L-serine and the experimental results was intriguing. In the experimental CD spectrum (10^−2^ M aqueous solution), a notable positive maximum of the Cotton effect was observed at around 200 nm [17]. The experimental CD spectrum is in agreement with our calculations for L-serine dimer in aqueous solution (Figure 11, black line, 197 nm) but not for the monomer (Figure 6 left, black line, near 145 nm). Obviously, L-serine, experimentally studied by Burkov and co-authors [17], was present in an aqueous solution in the form of dimers.

The contributions of the changes in Gibbs energy at room temperature were evaluated for two interactions in aqueous solution:


L-Ser + L-Ser → 2L-Ser − 25.21 kcal/mol
(2)

2L-Ser + 2L-Ser → 4L-Ser + 2.48 kcal/mol
(3)

According to computations at room temperature in aqueous solution, L-serine exists in the form of dimers. The first process (Equation (2), Δ*G* < 0) is spontaneous, whereas the second process (Equation (3), Δ*G* > 0) cannot occur.

### 2.5. Complexes of Four Serine Molecules with a Hydrogen Peroxide Molecule in the Gas Phase

Calculations were performed for complexes composed of four serine molecules in various configurations. X-ray analysis revealed that the H_2_O_2_ molecule forms four hydrogen bonds with four adjacent L-serine molecules, resulting in two donor and two acceptor interactions [18,19]. The perhydrate of L-serine was crystallized from a cooled aqueous solution of hydrogen peroxide saturated with serine.

One of the serine enzymes, bacteriophage TP901-1 integrase, crystallizes as a tetramer but is a dimer in solution [20].

**Figure 12:**
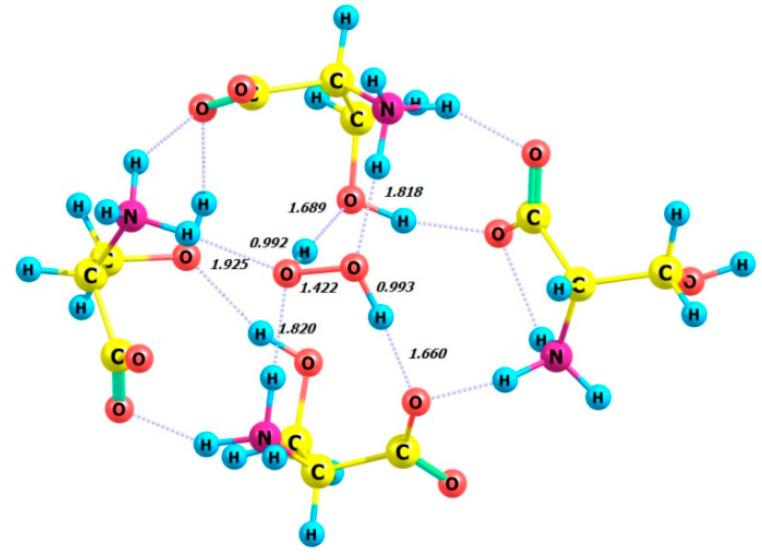
Structure of complex 4 L-Ser_M-H_2_O_2_.

**Figure 13:**
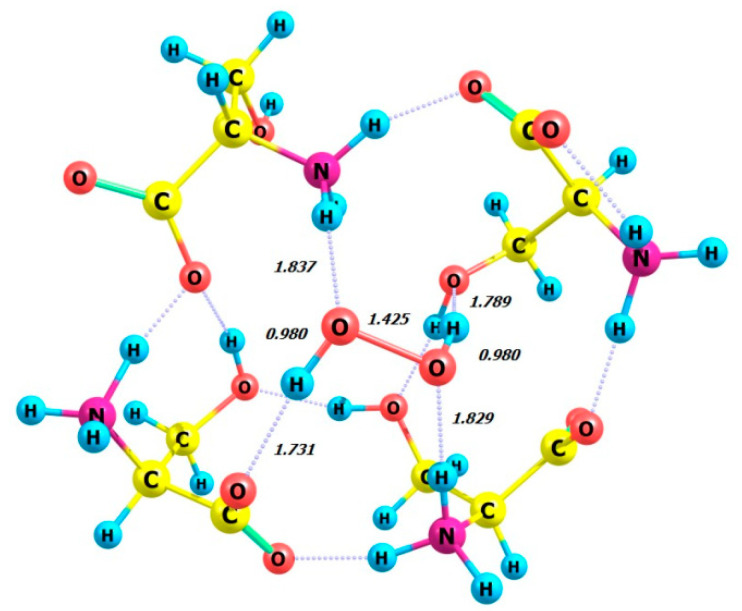
Structure of complex 4 L-Ser_P-H_2_O_2_.

**Figure 14:**
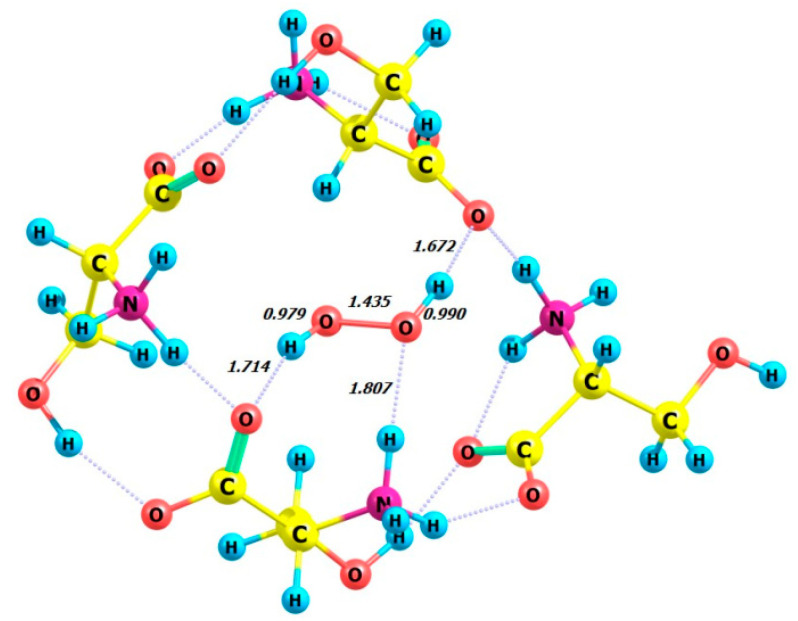
Structure of complex 4 D-Ser_M-H_2_O_2_.

**Figure 15:**
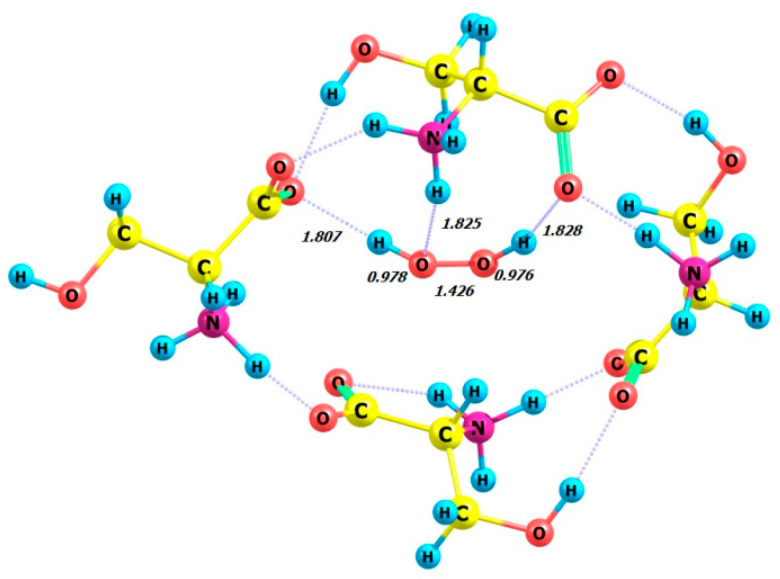
Structure of complex 4 D-Ser_P-H_2_O_2_.

The DFT calculation was performed on the model compound L-serine perhydrate, an analog from [18,19], using the ωb97xd/6-311+G** basis set. Calculations were performed on four complexes in the gas phase, each consisting of four enantiomeric serine molecules and either the P- or M-enantiomer of a hydrogen peroxide molecule. The complexes studied were 4 L-Ser_M-H_2_O_2_, 4 L-Ser_P-H_2_O_2_, 4 D-Ser_M-H_2_O_2_, and 4 D-Ser_P-H_2_O_2_. The optimized structures of these complexes are shown in Figure 12, Figure 13, Figure 14 and Figure 15, all of which exhibit the zwitterionic structure. Table 5 summarizes the HOOH dihedral angles and some energetic characteristics of hydrogen peroxide complexes in M and P forms with clusters of four molecules of L- and D-serine enantiomers.

Using the total energy values *E* from Table 5 for the complexes 4 L-Ser_M-H_2_O_2_, 4 L-Ser_P-H_2_O_2_, 4 D-Ser-M_H_2_O_2_, 4 D-Ser_P-H_2_O_2_, 4 L-Ser, and 4 D-Ser, the energies of non-covalent interactions of L- and D-serine tetramers with (M and P) hydrogen peroxide can be calculated for the gas phase and aqueous solution (Table 6):

Therefore, the formation of complexes 4 L-Ser_M-H_2_O_2_ and 4 L-Ser_P-H_2_O_2_ based on L-serine (Table 6, lines 1 and 2) is the most energetically favorable.

The structure of the complex 4 L-Ser_P-H_2_O_2_ (Figure 13) obtained from the calculations is similar to the structure of the crystal lattice fragment from [18]. The hydrogen peroxide molecule participates in four hydrogen bonds with the neighboring serine molecules. The ammonium groups act as hydrogen bond donors for the peroxide molecule, while the hydroxyl and carboxylate moieties accept hydrogen bonds from the peroxide. The HOOH dihedral angle was calculated for 4 L-Ser_P-H_2_O_2_ as equal to 114.29 degrees (Table 5, line 2), which closely matches the X-ray value of 109 (**2**) degrees [18].

**Figure 16:**
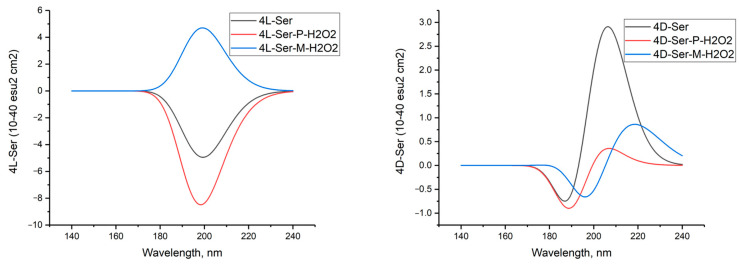
Circular dichroism spectra of hydrogen peroxide complexes in M and P forms with clusters of four molecules of L- and D-serine enantiomers in gas phase; DFT calculation method ωb97xd/6-311G**. A comparison of the CD spectra of these compounds obtained using three other DFT methods is given in the Appendix.

Figure 16 shows the CD spectra of 4L-Ser and 4L-Ser tetramers, as well as their complexes with P- and M-H_2_O_2_.

### 2.6. Complexes of Four Serine Molecules with a Hydrogen Peroxide Molecule in an Aqueous Solution

Table 7 summarizes the characteristics of the L and D-serine clusters and hydrogen peroxide molecule obtained by the same DFT method with the ωb97xd/6-311+G** basis set. The energy characteristics presented in Table 7 allowed us to write down the energetics of the following interactions (Table 6, column 3).

Calculations of these interactions suggest that hydrogen peroxide binds more strongly to the 4-L-Ser cluster than to the 4-D-Ser cluster, resulting in a significant energy gain.

Additionally, the average energy gain of hydrogen peroxide binding to the 4 L-Ser cluster is 13.95 kcal per mole. The value obtained in [10], 14 kcal per mole, practically coincides with this value.

**Figure 17:**
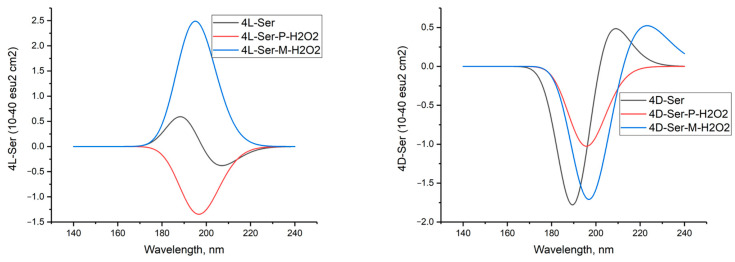
Circular dichroism spectra of hydrogen peroxide complexes in M and P forms with clusters of four molecules of L- and D-serine enantiomers in aqueous solution; DFT calculation method with the ωb97xd/6-311G** basis set. A comparison of the CD spectra of these compounds obtained using three other DFT methods is given in the Supplementary Materials.

In aqueous solution, all atoms of P-peroxide interact exhaustively with four molecules of L-serine, similar to the calculations for the complex of tetramer L-Ser with P-peroxide in the gas phase. The dihedral angle in peroxide, calculated to be 109.769 degrees (Table 7, second line), practically coincides with the angle determined experimentally by PCA, 109 (**2**) degrees [18]. CD spectra of 4L-Ser and 4L-Ser tetramers and their complexes with P- and M-H_2_O_2_ in aqueous solution are shown in Figure 17.

## 3. Calculation Methods

Two calculation methods were selected: the second-order Møller–Plesset theory (MP2) and the density functional theory (DFT) [21,22,23]. The Gaussian 09 program package [24] was used for all calculations with the ωb97xD functional [25], which includes the exact Hartree–Fock exchange, modified b97 exchange for short-range interaction, and Grimme dispersion corrections [26]. The DFT method used in this work, ωb97xD/6-311+G*, considers the van der Waals interaction energy. Other DFT methods, such as B3LYP [27], B3P86 [28], and M06 [29], were employed.

All calculations were performed while considering the effect of aqueous solvent. The continuum solvent model SMD [30] was used. The calculations were performed using the GAUSSIAN-09 program (version E.01) in the LINUX operating system, with full optimization of the molecule geometry and calculation of normal mode frequencies. The calculations were performed with increased accuracy, using the keywords “integral = (UltraFine, Acc2E = 12)”. Graphical representations were created using the ChemCraft program [31].

The transition states (TS) for the *cis*-*trans* transitions were calculated using the synchronous transit-directed quasi-Newton method with QST2 and QST3 options [32]. The IRC (internal reaction coordinate) method [33] was use to confirm the correspondence of the transition states obtained for the proposed interactions. This method has been successfully applied to calculate supramolecular systems in previous studies [34]. Circular dichroism spectra in the UV/vis region were calculated using the TD-DFT (time-dependent DFT) methodology [9]. The CD spectra calculated by the ωB97xD/6-31G(d) method for peptides were shown to be in good agreement with high-level ab initio RICC2 calculations [35].

## 4. Conclusions

Computational methods were used to assess homochirality at the microlevel in our research on hydrogen peroxide. Our main achievements relate to the interactions of hydrogen peroxide with amino acid enantiomers. The analysis of the interaction between each enantiomer of hydrogen peroxide (P and M) and the L- and D-serine enantiomers showed that hydrogen peroxide interacts more strongly with the dominant natural L-enantiomer of serine. The data were calculated for the gas phase and aqueous solution. It was found that solvation effects are significant in the studied processes of complex formation.

Computational studies confirmed that complexes based on the L-serine enantiomer have energetic advantages over D-serine in forming dimeric and tetrameric complexes with M- and P-peroxide. It is important to note that this statement is objective and does not include any subjective evaluations.

Circular dichroism (CD) spectra were simulated for all studied complexes for the first time. The calculated CD spectra provide a reliable means of assigning experimental CD spectra of the complexes.

The study examined the potential energy surfaces (PES) of M–P transitions between enantiomers of hydrogen peroxide in both the gas phase and aqueous solution. There are two barriers to the transition from one form (P) to another (M): a high barrier, TScis = TS (P-M), and a low barrier, TStrans = TS (P-M). Our calculations show that the activation energy for the gas phase *E* (TScis) is 7.21 kcal/mol, and the activation energy *E* (TStrans) is 1.26 kcal/mol, which correlate well with the experimental spectral results.

The peculiarities of hydrogen peroxide interactions with L-serine, an amino acid found in many natural proteins, have been revealed. This is an important step towards a deeper understanding of the underestimated role of hydrogen peroxide in both exogenous and endogenous emergence in the origin of life on Earth and the functioning of the biosphere at macro- and micro-levels.

## Figures and Tables

**Table 1:** Calculated total energy, *E*; dipole moment, μ; and HOMO and LUMO energies for L-serine and (L-Ser)*_n_* clusters, from *n* = 1 to *n* = 16, in the gaze phase by the DFT method with the ωb97xd/6-311+G* basis set.

*n*	*E*, a.u.	μ, D	HOMO, a.u.	LUMO, a.u.
1	−398.8337836	4.1518	−0.34101	0.08516
2	−797.6798965	2.4455	−0.33131	0.09125
3	−1196.5696345	2.9664	−0.31891	0.10479
4	−1595.427764	7.0445	−0.31052	0.10190
6	−2393.1876883	6.1978	−0.31462	0.08978
8	−3190.9895845	8.0066	−0.31578	0.08057
10	−3988.7061529	7.5975	−0.31582	0.06749
12	−4786.4338597	8.0780	−0.32131	0.06596
14	−5584.1619728	4.9998	−0.31480	0.06980
16	−6381.8954629	5.9206	−0.31508	0.07181

**Table 2:** Energy characteristics of hydrogen peroxide complexes in M and P forms with L- and D-serine enantiomers in the gas phase and in aqueous solution; MP2/aug-cc-pVDZ calculation method.

No	Complex	*E*(MP2), a.u.	HOMO, a.u.	LUMO, a.u.
**1**	L-Ser-M-H_2_O_2_	−549.2293614	−0.43598	0.02014
**2**	D-Ser-M-H_2_O_2_	−549.2267844	−0.44553	0.02009
**3**	L-Ser-P-H_2_O_2_	−549.2320886	−0.42693	0.02577
**4**	D-Ser-P-H_2_O_2_	−549.2283007	−0.43766	0.02749
**5**	L-Ser-M-H_2_O_2_/H_2_O	−549.2726099	−0.43096	0.03994
**6**	D-Ser-M-H_2_O_2_/H_2_O	−549.269142	−0.43224	0.04013
**7**	L-Ser-P-H_2_O_2_/H_2_O	−549.2729312	−0.43227	0.04068
**8**	D-Ser-P-H_2_O_2_/H_2_O	−549.2694565	−0.43404	0.04029

**Table 3:** Interactions energies Δ*E* of hydrogen peroxide complexes in M and P forms with L- and D-serine enantiomers in the gas phase and in aqueous solution; MP2/aug-cc-pVDZ calculation method.

No	Complex	Δ*E*(MP2), kcal/mol
**1**	L-Ser-M-H_2_O_2_	−10.43
**2**	D-Ser-M-H_2_O_2_	−8.81
**3**	L-Ser-P-H_2_O_2_	−12.14
**4**	D-Ser-P-H_2_O_2_	−9.76
**5**	L-Ser-M-H_2_O_2_/H_2_O	−10.91
**6**	D-Ser-M-H_2_O_2_/H_2_O	−8.73
**7**	L-Ser-P-H_2_O_2_/H_2_O	−11.11
**8**	D-Ser-P-H_2_O_2_/H_2_O	−8.93

**Table 4:** Structure and energy characteristics of L- and D-serine, their dimers, and complexes of two molecules of hydrogen peroxide in M form with L- and D-serine dimers in aqueous solution; DFT calculation method with the ωb97xd/6-311+G** basis set.

Complex	Dihedral HOOH Angles	*E*, a.u.	HOMO, a.u.	LUMO, a.u.	Δ*E*, kcal/mol
2 L-Ser_2 H_2_O_2_(MM)	−89.686 −92.237	−1101.187021	−0.35710	0.06506	−33.15
2 D-Ser_2 H_2_O_2_(MM)	−89.066 −97.170	−1101.1754803	−0.34637	0.06636	−29.24
2 L-Ser	−	−798.0269538	−0.35346	0.06376	−18.14 (2H_2_O_2_ MM)
2 D-Ser	−	−798.0207126	−0.34609	0.06404	−14.82 (2H_2_O_2_ MM)
L-Ser or D-Ser	−	−398.9870594	−0.34896	0.06487	−
H_2_O_2_	−97.079	−151.5655781	−0.39364	0.08984	−

**Table 5:** Structure and energetic characteristics (the total energies *E* and HOMO and LUMO energies) of hydrogen peroxide complexes in M and P forms with clusters of four molecules of L- and D-serine enantiomers in the gas phase; DFT method with the ωb97xd/6-311+G** basis set.

Complex	Dihedral HOOH Angle	*E*, a.u.	HOMO, a.u.	LUMO, a.u.
4 L-Ser_M-H_2_O_2_	−88.04	−1747.54748	−0.33028	0.04021
4 L-Ser_P-H_2_O_2_	114.29	−1747.54683	−0.32850	0.03422
4 D-Ser_M-H_2_O_2_	−162.15	−1747.55344	−0.34232	0.04288
4 D-Ser_P-H_2_O_2_	133.38	−1747.54856	−0.33931	0.04293
4 L-Ser	−	−1595.96141	−0.32207	0.04472
4 D-Ser	−	−1595.97878	−0.33906	0.03984
H_2_O_2_	−119.09	−151.54948	−0.37504	0.08309

**Table 6:** The energy changes Δ*E* for the interactions of hydrogen peroxide complexes in M and P forms with clusters of four molecules of L- and D-serine enantiomers in the gas phase and in aqueous solution; DFT method with the ωb97xd/6-311+G** basis set.

Complex	Δ*E*, kcal/mol, Gas Phase	Δ*E*, kcal/mol, Aqueous Solution
4 L-Ser_M-H_2_O_2_	−22.96	−12.89
4 L-Ser_P-H_2_O_2_	−22.55	−15.01
4 D-Ser_M-H_2_O_2_	−15.74	−8.25
4 D-Ser_P-H_2_O_2_	−12.74	−6.51

**Table 7:** Structure and energy characteristics of hydrogen peroxide complexes in M and P forms with clusters of four molecules of L- and D-serine enantiomers in aqueous solution; DFT calculation method ωb97xd/6-311+G**.

Complex	Dihedral HOOH Angle	*E*, a.u.	HOMO, a.u.	LUMO, a.u.
4 L-Ser_M_H_2_O_2_	−83.153	−1747.6606928	−0.34978	0.06214
4 L-Ser_P_H_2_O_2_	109.769	−1747.66408063	−0.34937	0.06173
4 D-Ser_M_H_2_O_2_	−164.026	−1747.6584351	−0.34528	0.06505
4 D-Ser_P_H_2_O_2_	119.169	−1747.655658	−0.35018	0.06481
4 L-Ser	−	−1596.074579	−0.34844	0.06130
4 D-Ser	−	−1596.079713	−0.35189	0.06226
H_2_O_2_	−97.079	−151.5655781	−0.39364	0.08984

## Data Availability

All data that supports the findings of this study is available in the published article and the Supplementary Materials to this article.

## Supplementary Materials

The following supporting information can be downloaded at: https://www.mdpi.com/article/10.3390/molecules29163955/s1, Figure S1: Dependence of the total energy MP2/aug-cc-pVDZ on the NOON dihedral angle for the H_2_O_2_-H_2_O_2_ dimer in aqueous solution for the continuum solvent SMD model; Figure S2: Structure of a cluster of four L-serine molecules, the DFT method with the ωb97xd basis set; Figure S3: Structure of a cluster of eight L-serine molecules, the DFT method with the ωb97xd basis set; Figure S4. Circular dichroism spectra of complexes of two molecules of hydrogen peroxide in M form with clusters of two molecules of D-enantiomers of serine in aqueous solution obtained using different DFT methods; Figure S5. Circular dichroism spectra of clusters of two molecules of L-enantiomers of serine in aqueous solution obtained using different DFT methods; Figure S6. Circular dichroism spectra of complexes of two molecules of hydrogen peroxide in M form with clusters of two molecules of L-enantiomers of serine in aqueous solution obtained using different DFT methods; Figure S7. Circular dichroism spectra of clusters of two molecules of D-enantiomers of serine in aqueous solution obtained using different DFT methods; Figure S8. Calculated CD spectra of hydrogen peroxide in M and P forms and its tetrameric clusters; MP2/6-311+G**; Table S1: Geometric structure of hydrogen peroxide dimers, MP2/6-311+G**; Table S2: Geometric structure of hydrogen peroxide clusters, MP2/6-311+G**; Table S3: Cartesian coordinates of atoms (XYZ, in angstroms) for L- and D-serine dimers, DFT calculation method with the ωb97xd/6-311+G* basis set; Table S4: Cartesian coordinates of atoms (XYZ, in angstroms) for the L-serine dimer and two hydrogen peroxide (MM) molecules in aqueous solution, DFT calculation method with the ωb97xd/6-311+G* basis set; Table S5: Cartesian coordinates of atoms (XYZ, in angstroms) of 4 L-Ser_H_2_O_2_ complexes, DFT calculation method ωb97xd/6-311+G*; Table S6: Cartesian coordinates of atoms (XYZ, in angstroms) of 4 D-Ser_H_2_O_2_ complexes, DFT calculation method with the ωb97xd/6-311+G* basis set; Table S7: Cartesian coordinates of atoms (XYZ, in angstroms) for a cluster of four L-serine molecules, DFT calculation method ωb97xd/6-311+G*; Table S8: Cartesian coordinates of atoms (XYZ, in angstroms) for a cluster of eight L-serine molecules, DFT calculation method with the ωb97xd/6-31G* basis set; Table S9: Results of calculations of L serine, n-Ser-L, clusters from n = 1 to n = 16 by the DFT B3LYP method; Table S10: Results of calculations of L serine, n-Ser-L, clusters from n = 1 to n = 16 by the B3P86 DFT method; Table S11: Results of calculations of L serine, n-Ser-L, clusters from n = 1 to n = 16 by the DFT M06 method; Table S12. Calculated bond lengths and bond angles for the complexes 1–8.
