# Supplementary material for: Complexes of Hydrogen Peroxide, the Simplest Chiral Molecule, with L- and D-Serine Enantiomers and Their Clusters: MP2 and DFT Calculations
Source: Molecules. 2024 Aug 21;29(16):3955. doi: 10.3390/molecules29163955 (PMC11356864; doi:10.3390/molecules29163955)
Supplement: Supplementary file 1 [file molecules-29-03955-s001.zip › molecules-3093715-supplementary-final_1 1.pdf]

SUPPORTING MATERIALS

Complexes of Hydrogen Peroxide, the Simplest Chiral Molecule, with L- and D-Serine Enantiomers and Their Clusters: MP2 and DFT Calculations[1]

Yurii A. Borisov [1], Sergey S. Kiselev [1], Mikhayl I. Budnik [2], Lubov V. Snegur [1]*

Isomerization of H_2O_2-H_2O_2 dimer in aqueous solution for the continuum solvent SMD model

Figure S1 shows the dependence of the MP2/aug-cc-pVDZ total energy on the NOON dihedral angle for the H_2O_2-H_2O_2 dimer in aqueous solution for the continuum SMD solvent model.

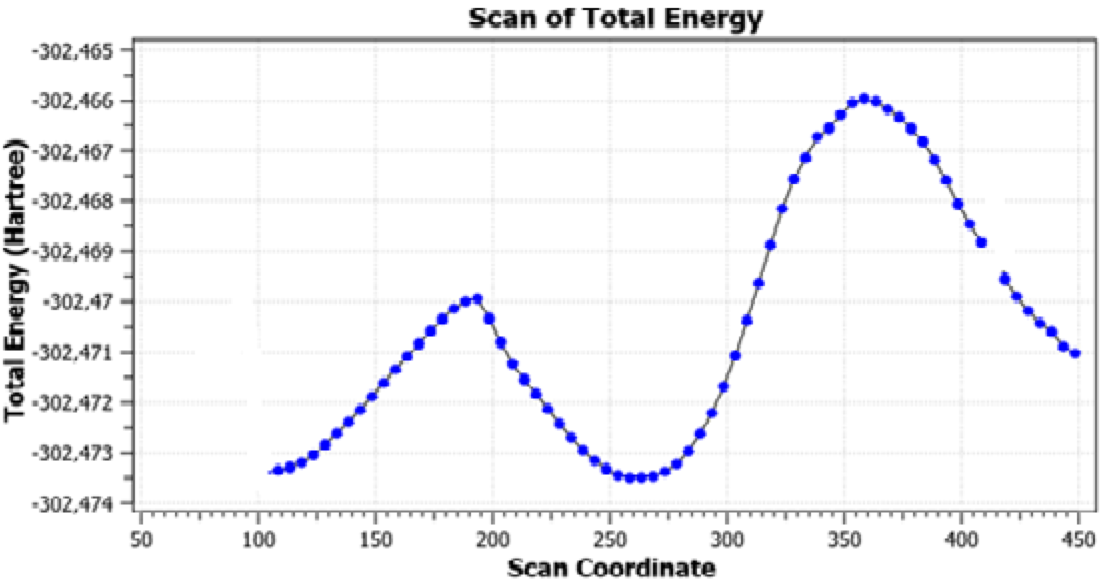

Figure S1. Dependence of the total energy MP2/aug-cc-pVDZ on the NOON dihedral angle for the H_2O_2-H_2O_2 dimer in aqueous solution for the continuum solvent SMD model.

In this case, the activation energy for *trans*-orientation is 2.28 kcal/mol and for *cis*-orientation 4.77 kcal/mol.

[1] Dedicated to the memory of Professor V.A. Davankov.

Structure of dimers, cyclic trimer and cyclic tetramers (M-M, M-P, P-P, M-M-M, M-M-M-M, M-P-M-P, P-P-P-P)

Table S1. Geometric structure of hydrogen peroxide dimers, MP2/6-311+G**.

<table>
<tr><th>Hydrogen peroxide clusters</th><th>Structure</th></tr>
<tr><td>M-M</td><td>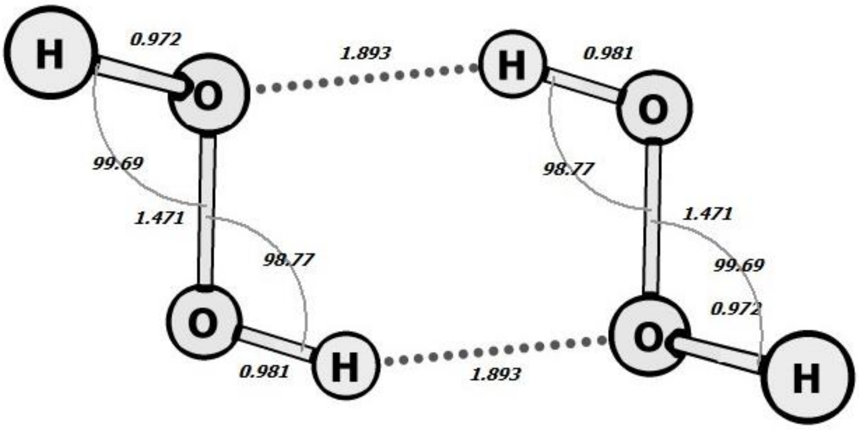
</td></tr>
<tr><td>M-P</td><td>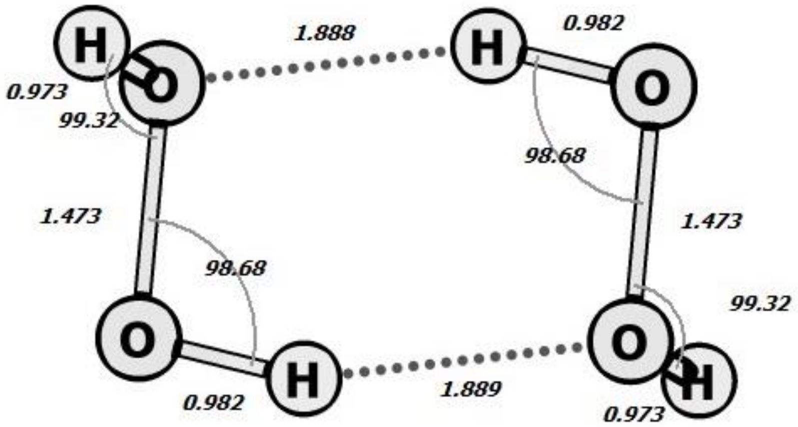
</td></tr>
<tr><td>P-P</td><td>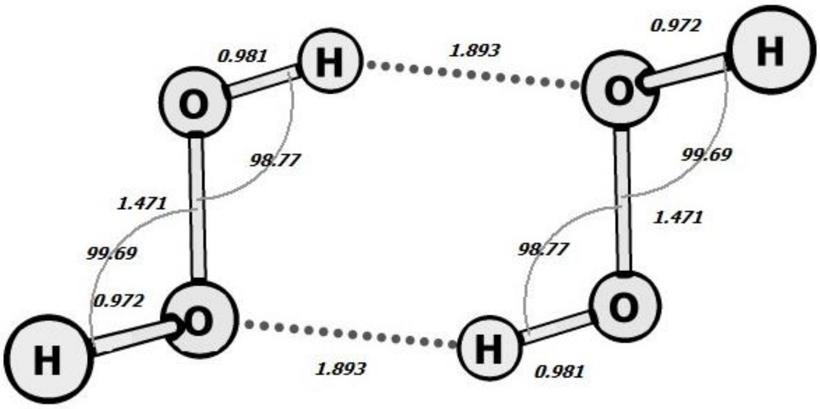
</td></tr>
</table>

Table S2. Geometric structure of hydrogen peroxide clusters, MP2/6-311+G**.

M-M-M	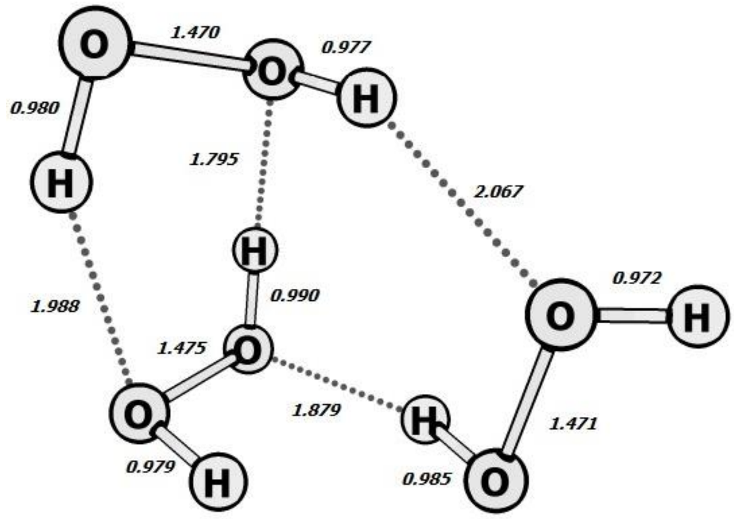
M-M-M-M	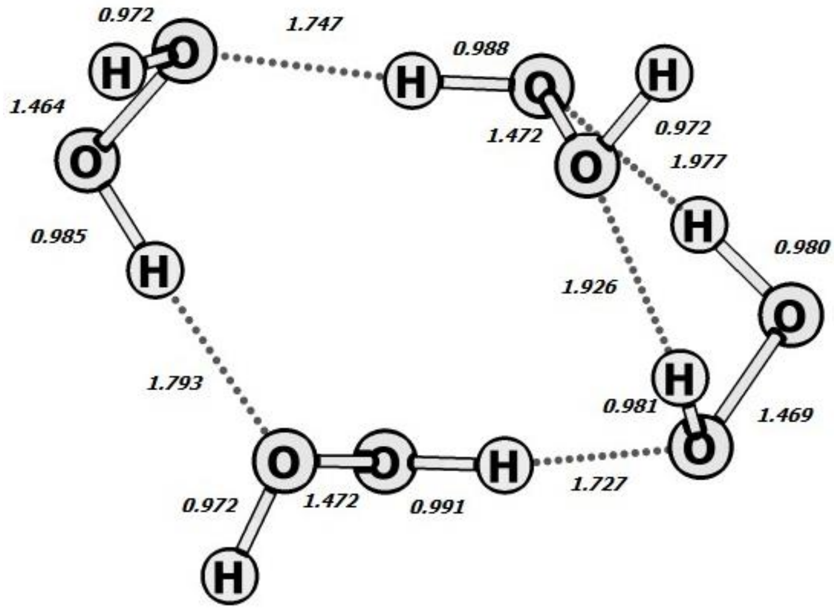
M-P-M-P	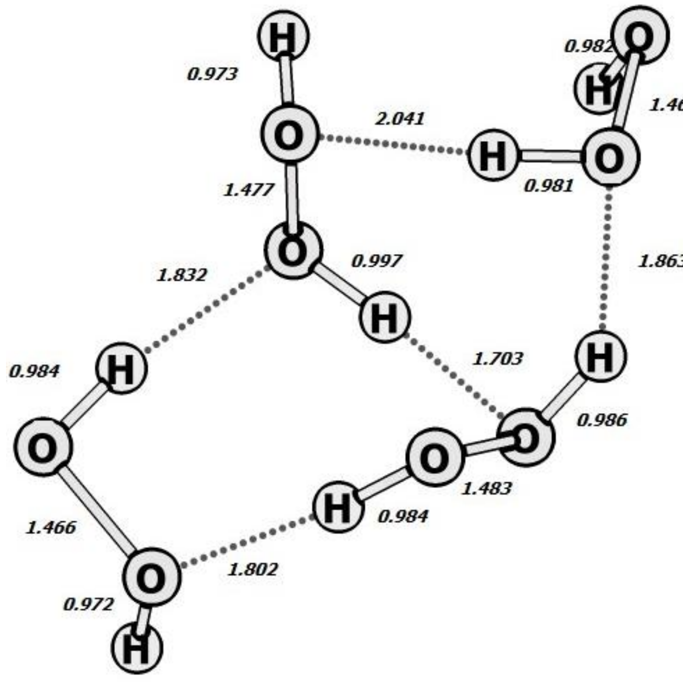

P-P-P-P	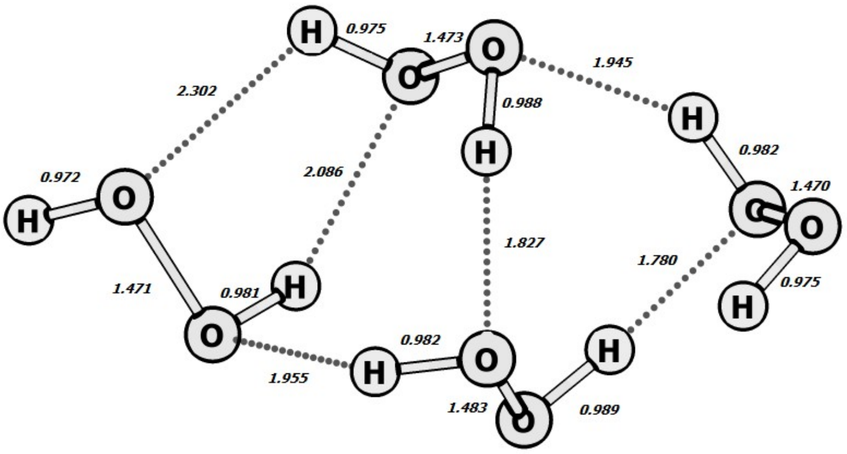

Table S3. Cartesian coordinates of atoms (XYZ, in angstroms) for L- and D-serine dimers, DFT calculation method with the ωb97xd/6-311+G* basis set.

Dimer of L-serine in aqueous solution				Dimer of D-serine in aqueous solution			
**7**	**-1.470811000**	**-0.583780000**	**-1.586049000**	**7**	**3.503802000**	**0.521298000**	**-0.582132000**
**8**	**-1.263172000**	**-1.679474000**	**1.255564000**	**6**	**2.135073000**	**0.216367000**	**-0.078537000**
**8**	**-2.162306000**	**1.150950000**	**1.442647000**	**1**	**1.451172000**	**0.369014000**	**-0.913817000**
**8**	**-1.438255000**	**1.882707000**	**-0.546557000**	**1**	**3.603970000**	**1.517679000**	**-0.786864000**
**6**	**-1.949305000**	**1.026928000**	**0.220231000**	**1**	**4.201521000**	**0.252988000**	**0.115994000**
**6**	**-2.378187000**	**-0.301141000**	**-0.440349000**	**6**	**1.775576000**	**1.170690000**	**1.043659000**
**1**	**-3.373450000**	**-0.135784000**	**-0.860869000**	**1**	**0.908475000**	**0.775793000**	**1.576415000**
**6**	**-2.455973000**	**-1.479705000**	**0.515011000**	**1**	**2.608734000**	**1.259082000**	**1.748470000**
**1**	**-3.307576000**	**-1.320637000**	**1.181445000**	**8**	**1.475900000**	**2.435944000**	**0.460063000**
**1**	**-2.635479000**	**-2.396547000**	**-0.049992000**	**1**	**1.388213000**	**3.085560000**	**1.163888000**
**1**	**-0.537660000**	**-0.962442000**	**-1.288501000**	**6**	**2.051579000**	**-1.251171000**	**0.379300000**
**1**	**-1.158037000**	**-0.914714000**	**1.832601000**	**8**	**3.121383000**	**-1.870158000**	**0.533282000**
**1**	**-1.891828000**	**-1.253648000**	**-2.229100000**	**8**	**0.887739000**	**-1.685117000**	**0.569769000**
**7**	**2.538385000**	**0.496768000**	**1.368084000**	**7**	**-1.197468000**	**-0.876550000**	**-1.129362000**
**8**	**1.217674000**	**2.524089000**	**-0.271436000**	**6**	**-2.313239000**	**-0.039220000**	**-0.618923000**
**8**	**1.061948000**	**-1.655767000**	**-1.127121000**	**1**	**-2.931783000**	**0.222324000**	**-1.479623000**
**8**	**3.023480000**	**-1.716406000**	**-0.046808000**	**1**	**-0.694006000**	**-0.371779000**	**-1.860513000**
**6**	**1.935430000**	**-1.189908000**	**-0.350991000**	**1**	**-0.503628000**	**-1.122214000**	**-0.387187000**
**6**	**1.606125000**	**0.182065000**	**0.251843000**	**6**	**-1.749803000**	**1.227145000**	**0.010916000**
**1**	**0.604910000**	**0.146326000**	**0.679779000**	**1**	**-2.588080000**	**1.858715000**	**0.313169000**
**6**	**1.681010000**	**1.293500000**	**-0.792348000**	**1**	**-1.176508000**	**0.966537000**	**0.905712000**
**1**	**2.719493000**	**1.435089000**	**-1.103985000**	**8**	**-0.928523000**	**1.903715000**	**-0.928331000**
**1**	**1.098271000**	**0.987997000**	**-1.664916000**	**1**	**-0.113620000**	**2.183533000**	**-0.478960000**
**1**	**2.406024000**	**1.461027000**	**1.681347000**	**6**	**-3.172136000**	**-0.834092000**	**0.379684000**
**1**	**2.384984000**	**-0.124734000**	**2.163233000**	**8**	**-2.811890000**	**-2.002252000**	**0.649687000**
**1**	**-1.293053000**	**0.291027000**	**-2.085675000**	**8**	**-4.168626000**	**-0.227044000**	**0.835315000**
**1**	**0.244148000**	**2.458140000**	**-0.240617000**	**1**	**-1.553407000**	**-1.748966000**	**-1.519093000**
**1**	**3.507708000**	**0.376262000**	**1.064562000**	**1**	**3.705417000**	**-0.003739000**	**-1.434150000**

Table S4. Cartesian coordinates of atoms (XYZ, in angstroms) for the L-serine dimer and two hydrogen peroxide (MM) molecules in aqueous solution, DFT calculation method with the ωb97xd/6-311+G* basis set.

**Dimer of L-serine + two molecules of hydrogen peroxide (MM) in aqueous solution**				**Dimer of D-serine + two molecules of hydrogen peroxide (MM) in aqueous solution**			
**7**	**1.196076000**	**1.198406000**	**-1.306976000**	**7**	**2.589036000**	**0.142306000**	**-0.026373000**
**8**	**-1.254143000**	**2.007949000**	**0.350902000**	**6**	**1.141464000**	**0.097178000**	**-0.367148000**
**8**	**0.830408000**	**1.528633000**	**2.215420000**	**1**	**0.602128000**	**-0.064735000**	**0.566786000**
**8**	**2.286936000**	**0.281160000**	**1.080823000**	**1**	**2.771855000**	**0.912260000**	**0.639151000**
**6**	**1.443018000**	**1.213975000**	**1.179593000**	**1**	**2.897288000**	**-0.749533000**	**0.389522000**
**6**	**1.157730000**	**2.049096000**	**-0.086304000**	**6**	**0.834823000**	**-1.041632000**	**-1.327688000**
**1**	**1.975875000**	**2.767871000**	**-0.179655000**	**1**	**-0.117475000**	**-0.840981000**	**-1.819960000**
**6**	**-0.149717000**	**2.824819000**	**-0.010528000**	**1**	**1.617601000**	**-1.117632000**	**-2.088711000**
**1**	**-0.028375000**	**3.640759000**	**0.704973000**	**8**	**0.742889000**	**-2.266624000**	**-0.590187000**
**1**	**-0.379714000**	**3.256609000**	**-0.985718000**	**1**	**0.533999000**	**-2.976002000**	**-1.207143000**
**1**	**0.380588000**	**0.547220000**	**-1.375177000**	**6**	**0.692815000**	**1.445699000**	**-0.963766000**
**1**	**-1.104841000**	**1.711507000**	**1.257185000**	**8**	**1.582845000**	**2.234142000**	**-1.358196000**
**1**	**1.181365000**	**1.789780000**	**-2.138607000**	**8**	**-0.543170000**	**1.629945000**	**-1.021874000**
**1**	**-3.730514000**	**-0.447902000**	**-0.143501000**	**1**	**2.211456000**	**-2.746004000**	**0.337888000**
**8**	**-4.082604000**	**0.361111000**	**0.278226000**	**8**	**3.081512000**	**-2.665179000**	**0.784251000**
**8**	**-3.719625000**	**1.377273000**	**-0.655593000**	**8**	**3.996697000**	**-3.012986000**	**-0.249866000**
**1**	**-2.831424000**	**1.651148000**	**-0.343202000**	**1**	**4.174613000**	**-3.945610000**	**-0.072695000**
**7**	**-1.600028000**	**-2.205590000**	**1.326593000**	**7**	**-2.167763000**	**0.804309000**	**1.125324000**
**8**	**1.318723000**	**-2.301323000**	**1.315632000**	**6**	**-3.163153000**	**-0.265474000**	**0.857275000**
**8**	**-0.911557000**	**-0.567730000**	**-1.827122000**	**1**	**-3.624999000**	**-0.518059000**	**1.813581000**
**8**	**-2.689417000**	**-1.672331000**	**-1.035281000**	**1**	**-1.498104000**	**0.487377000**	**1.827904000**
**6**	**-1.522260000**	**-1.223389000**	**-0.953247000**	**1**	**-1.627716000**	**1.058247000**	**0.268464000**
**6**	**-0.751407000**	**-1.472471000**	**0.348286000**	**6**	**-2.452096000**	**-1.485589000**	**0.290087000**
**1**	**-0.533533000**	**-0.503220000**	**0.797873000**	**1**	**-3.192023000**	**-2.276993000**	**0.151545000**
**6**	**0.551789000**	**-2.234871000**	**0.128716000**	**1**	**-2.025546000**	**-1.237547000**	**-0.686912000**
**1**	**0.333601000**	**-3.259952000**	**-0.182182000**	**8**	**-1.432111000**	**-1.904682000**	**1.184001000**
**1**	**1.105143000**	**-1.741419000**	**-0.673180000**	**1**	**-0.644592000**	**-2.114820000**	**0.656976000**
**1**	**-1.059078000**	**-2.428372000**	**2.165227000**	**6**	**-4.255857000**	**0.243628000**	**-0.099080000**
**1**	**-2.409454000**	**-1.645615000**	**1.598850000**	**8**	**-4.139535000**	**1.409070000**	**-0.540656000**
**1**	**3.215334000**	**-1.454755000**	**-1.682636000**	**8**	**-5.173581000**	**-0.571161000**	**-0.349351000**
**8**	**3.529642000**	**-0.540561000**	**-1.691821000**	**1**	**2.100323000**	**2.853398000**	**1.955358000**
**8**	**4.303520000**	**-0.446508000**	**-0.499215000**	**8**	**2.911524000**	**2.564333000**	**1.516124000**
**1**	**3.628376000**	**-0.122154000**	**0.142750000**	**8**	**3.095201000**	**3.530837000**	**0.487392000**
**1**	**2.048857000**	**0.619543000**	**-1.350942000**	**1**	**2.589745000**	**3.135904000**	**-0.258354000**
**1**	**1.716139000**	**-1.419634000**	**1.436573000**	**1**	**-2.628365000**	**1.647237000**	**1.467210000**
**1**	**-1.950678000**	**-3.075478000**	**0.919177000**	**1**	**3.144749000**	**0.322773000**	**-0.864442000**

Table S5. Cartesian coordinates of atoms (XYZ, in angstroms) of 4 L-Ser_H_2O_2 complexes, DFT calculation method ωb97xd/6-311+G*.

4 L-Ser_M-H_2O_2				4 L-Ser_P-H_2O_2			
8	0.229531000	-0.293663000	-1.514279000	8	0.214048000	0.244338000	-2.044217000
8	-0.922574000	-0.117452000	-0.699527000	8	-1.057418000	-0.359537000	-1.823565000
1	0.689634000	0.577086000	-1.387596000	1	-1.346117000	0.063314000	-0.987938000
1	-0.613648000	-0.422604000	0.192230000	7	-1.211768000	-3.061476000	-0.873569000
7	-3.622254000	-1.135412000	-0.962056000	8	-0.451764000	-1.663287000	1.509040000
8	-2.657589000	-0.216188000	1.591106000	8	-3.726474000	-1.966527000	1.483825000
8	-3.821960000	2.285191000	-0.041101000	8	-3.317034000	-1.521517000	-0.680468000
8	-3.487967000	1.243591000	-1.996322000	6	-3.133380000	-2.118473000	0.420082000
6	-3.850035000	1.296391000	-0.811051000	6	-1.973204000	-3.153658000	0.409149000
6	-4.315659000	-0.051883000	-0.197345000	1	-2.403118000	-4.156001000	0.477608000
1	-5.391109000	-0.160524000	-0.363898000	6	-1.033103000	-2.950411000	1.588459000
6	-4.038274000	-0.190861000	1.295386000	1	-1.629679000	-3.036482000	2.499264000
1	-4.473154000	0.681662000	1.782703000	1	-0.250102000	-3.713104000	1.585578000
1	-4.519567000	-1.093909000	1.687157000	1	-1.135274000	-2.071557000	-1.183907000
1	-2.648884000	-0.842491000	-1.146330000	1	0.519920000	-1.687795000	1.589646000
1	-2.353979000	-1.139250000	1.555805000	1	-0.235050000	-3.437333000	-0.774779000
1	-3.537180000	-2.024225000	-0.431567000	1	-1.705276000	-3.534808000	-1.624739000
1	-4.042419000	-1.280449000	-1.875350000	7	0.555297000	2.402922000	-0.194746000
7	3.733531000	1.361578000	-0.155259000	8	1.835979000	3.290697000	2.082503000
8	6.056602000	0.125799000	0.573378000	8	4.133911000	2.362841000	-0.541120000
8	3.374604000	-2.177955000	-0.510469000	8	2.619068000	0.813178000	0.020007000
8	2.742991000	-0.664692000	1.031036000	6	3.003144000	2.007544000	-0.258174000
6	3.339518000	-1.043940000	0.002335000	6	1.875907000	3.060422000	-0.263668000
6	4.111363000	0.062150000	-0.764730000	1	1.937452000	3.609838000	-1.204288000
1	3.766219000	0.050165000	-1.800176000	6	2.012191000	4.037638000	0.891411000
6	5.614509000	-0.147654000	-0.741150000	1	3.006522000	4.492466000	0.839709000
1	5.806402000	-1.182558000	-1.040855000	1	1.247566000	4.818421000	0.799812000
1	6.099990000	0.525051000	-1.462419000	1	0.465232000	1.880530000	0.677770000
1	3.485108000	1.159895000	0.823517000	1	1.732746000	3.887663000	2.822903000
1	6.963292000	-0.161563000	0.677153000	1	-0.262396000	3.066578000	-0.203402000
1	4.458679000	2.073092000	-0.183022000	1	0.437720000	1.698242000	-0.943475000
1	2.841770000	1.737417000	-0.591402000	7	-4.087384000	0.997483000	-0.731183000
7	-1.305520000	2.708188000	-0.851576000	8	-1.491080000	0.629975000	0.702450000
8	-0.541709000	1.639981000	1.763679000	8	-1.807165000	3.634699000	0.128325000
8	1.941410000	3.622866000	0.436666000	8	-2.580494000	2.738470000	-1.784122000
8	1.303845000	2.079973000	-1.040678000	6	-2.532947000	2.878743000	-0.553477000
6	1.082182000	3.010311000	-0.185406000	6	-3.455745000	1.915670000	0.264673000
6	-0.384955000	3.382486000	0.093793000	1	-4.234440000	2.500378000	0.757265000
1	-0.493868000	4.461868000	-0.031834000	6	-2.655633000	1.168904000	1.315993000
6	-0.775119000	3.003742000	1.526884000	1	-2.368992000	1.889884000	2.086039000
1	-0.145941000	3.594437000	2.196941000	1	-3.251418000	0.366322000	1.762106000
1	-1.823980000	3.271367000	1.697347000	1	-3.729068000	1.379099000	-1.636023000
1	-1.174324000	1.682787000	-0.799359000	1	-1.207511000	-0.193131000	1.150409000
1	-1.369266000	1.136190000	1.796632000	1	-3.806097000	-0.038924000	-0.644668000
1	-2.321384000	2.853492000	-0.607569000	1	-5.100668000	1.044463000	-0.714940000
1	-1.146845000	2.968676000	-1.821009000	7	3.929925000	-1.153505000	-0.927553000
7	0.813569000	-2.981002000	-0.752113000	8	2.222528000	-1.276342000	1.669549000

**8**	**0.190615000**	**-1.215940000**	**1.447153000**	**8**	**1.404691000**	**-3.634768000**	**-0.311695000**
**8**	**-2.376967000**	**-2.863678000**	**0.771073000**	**8**	**1.687516000**	**-2.014931000**	**-1.834687000**
**8**	**-1.716177000**	**-3.365642000**	**-1.308062000**	**6**	**2.028050000**	**-2.701308000**	**-0.847291000**
**6**	**-1.527803000**	**-3.194407000**	**-0.095764000**	**6**	**3.397501000**	**-2.350206000**	**-0.214882000**
**6**	**-0.063885000**	**-3.380805000**	**0.385879000**	**1**	**4.077658000**	**-3.188678000**	**-0.388767000**
**1**	**0.098994000**	**-4.447670000**	**0.565388000**	**6**	**3.288957000**	**-2.115363000**	**1.292430000**
**6**	**0.298529000**	**-2.610563000**	**1.643508000**	**1**	**3.106539000**	**-3.083470000**	**1.758284000**
**1**	**-0.394047000**	**-2.917628000**	**2.429382000**	**1**	**4.239841000**	**-1.724529000**	**1.678519000**
**1**	**1.319491000**	**-2.855576000**	**1.953290000**	**1**	**3.460937000**	**-0.232212000**	**-0.526863000**
**1**	**0.630834000**	**-1.995870000**	**-1.018884000**	**1**	**2.339053000**	**-0.385950000**	**1.288983000**
**1**	**1.100122000**	**-0.852397000**	**1.426359000**	**1**	**4.940809000**	**-1.067956000**	**-0.876928000**
**1**	**1.839798000**	**-3.003240000**	**-0.550141000**	**1**	**3.613869000**	**-1.214808000**	**-1.899289000**
**1**	**0.554516000**	**-3.534022000**	**-1.568719000**	**1**	**0.815918000**	**-0.523732000**	**-1.949377000**

Table S6: Cartesian coordinates of atoms (XYZ, in angstroms) of 4 D-Ser_H_2O_2 complexes, DFT calculation method with the ωb97xd/6-311+G* basis set.

4 D-Ser_M-H_2O_2				4 D-Ser_P-H_2O_2			
**8**	**-0.511398000**	**0.083707000**	**-1.232532000**	**8**	**-0.454459000**	**-0.097953000**	**-0.009059000**
**8**	**0.389585000**	**-0.035172000**	**-0.121378000**	**8**	**0.727613000**	**0.028882000**	**0.778965000**
**1**	**1.060811000**	**-0.616440000**	**-0.534442000**	**1**	**1.340789000**	**-0.481149000**	**0.216442000**
**7**	**1.295262000**	**3.652387000**	**1.153100000**	**7**	**0.845335000**	**3.084242000**	**1.330137000**
**6**	**0.481819000**	**3.024897000**	**0.074867000**	**6**	**0.106413000**	**2.936989000**	**0.043225000**
**1**	**0.884692000**	**2.024937000**	**-0.077648000**	**1**	**0.518374000**	**2.055771000**	**-0.445187000**
**1**	**1.707162000**	**4.523908000**	**0.818370000**	**1**	**1.002314000**	**4.066610000**	**1.545987000**
**1**	**0.655840000**	**3.817891000**	**1.942245000**	**1**	**0.221734000**	**2.669832000**	**2.039173000**
**1**	**2.095237000**	**2.991398000**	**1.377148000**	**1**	**1.791890000**	**2.606066000**	**1.303044000**
**6**	**0.636105000**	**3.829034000**	**-1.219333000**	**6**	**0.344710000**	**4.150527000**	**-0.846114000**
**1**	**0.233135000**	**3.231358000**	**-2.042603000**	**1**	**-0.085641000**	**3.938077000**	**-1.829686000**
**1**	**0.066207000**	**4.761985000**	**-1.165247000**	**1**	**-0.167761000**	**5.031144000**	**-0.443840000**
**8**	**1.982759000**	**4.184583000**	**-1.412591000**	**8**	**1.714548000**	**4.467423000**	**-0.903693000**
**1**	**2.517831000**	**3.367514000**	**-1.384920000**	**1**	**2.218336000**	**3.655503000**	**-1.094163000**
**6**	**-0.960965000**	**2.877031000**	**0.565723000**	**6**	**-1.358567000**	**2.614283000**	**0.375082000**
**8**	**-1.269359000**	**3.372281000**	**1.648183000**	**8**	**-1.631782000**	**2.383058000**	**1.565860000**
**8**	**-1.718242000**	**2.213150000**	**-0.216454000**	**8**	**-2.151193000**	**2.559608000**	**-0.603582000**
**7**	**4.148934000**	**-0.664220000**	**-1.127449000**	**7**	**4.151505000**	**-0.349193000**	**-1.445499000**
**6**	**4.279385000**	**-0.031856000**	**0.219645000**	**6**	**4.445419000**	**0.282772000**	**-0.126868000**
**1**	**5.337188000**	**0.209738000**	**0.357381000**	**1**	**5.465456000**	**0.673323000**	**-0.177546000**
**1**	**3.263732000**	**-1.254842000**	**-1.191727000**	**1**	**3.299191000**	**-0.975290000**	**-1.342209000**
**1**	**4.061465000**	**0.111953000**	**-1.795596000**	**1**	**3.911773000**	**0.418796000**	**-2.083305000**
**1**	**4.940826000**	**-1.267957000**	**-1.329875000**	**1**	**4.931922000**	**-0.914496000**	**-1.767803000**
**6**	**3.857677000**	**-1.029678000**	**1.294509000**	**6**	**4.351871000**	**-0.779732000**	**0.971636000**
**1**	**4.113612000**	**-0.609653000**	**2.268481000**	**1**	**4.843460000**	**-0.393980000**	**1.866340000**
**1**	**2.770675000**	**-1.168058000**	**1.273551000**	**1**	**3.305178000**	**-0.961805000**	**1.232038000**
**8**	**4.537955000**	**-2.243915000**	**1.088240000**	**8**	**4.981668000**	**-1.962103000**	**0.534352000**
**1**	**3.897135000**	**-2.901505000**	**0.761777000**	**1**	**4.295043000**	**-2.641408000**	**0.405974000**
**6**	**3.501961000**	**1.299894000**	**0.186646000**	**6**	**3.481026000**	**1.481233000**	**0.016690000**
**8**	**3.277473000**	**1.759087000**	**-0.956050000**	**8**	**3.050249000**	**1.951942000**	**-1.060783000**
**8**	**3.196964000**	**1.785462000**	**1.294051000**	**8**	**3.228331000**	**1.847848000**	**1.178671000**
**7**	**-3.844507000**	**1.055157000**	**0.794730000**	**7**	**-4.039921000**	**1.085640000**	**0.346752000**

6	-4.224133000	0.111051000	-0.286973000	6	-4.356827000	-0.074619000	-0.515226000
1	-3.961618000	0.566032000	-1.241926000	1	-4.308782000	0.252624000	-1.553850000
1	-4.623629000	1.666239000	1.037133000	1	-4.888284000	1.613050000	0.550806000
1	-3.544827000	0.470819000	1.587762000	1	-3.609268000	0.754023000	1.216441000
1	-3.004331000	1.640494000	0.476258000	1	-3.290386000	1.763460000	-0.106046000
6	-5.717348000	-0.165968000	-0.230092000	6	-5.755187000	-0.588024000	-0.200884000
1	-5.993339000	-0.747321000	-1.113698000	1	-6.016466000	-1.354844000	-0.935416000
1	-5.951649000	-0.752417000	0.667998000	1	-5.767834000	-1.034970000	0.801822000
8	-6.370432000	1.094315000	-0.192088000	8	-6.626315000	0.529963000	-0.265563000
1	-7.316258000	0.965956000	-0.118301000	1	-7.512467000	0.264045000	-0.019832000
6	-3.434152000	-1.201528000	-0.078955000	6	-3.331467000	-1.199626000	-0.280551000
8	-2.975077000	-1.360047000	1.074832000	8	-2.736198000	-1.175089000	0.833469000
8	-3.359167000	-1.965118000	-1.055996000	8	-3.195414000	-2.033816000	-1.185665000
7	-0.784855000	-2.743458000	-1.296900000	7	-0.526213000	-2.620996000	-1.230840000
6	0.129761000	-3.458467000	-0.360581000	6	0.370008000	-3.415433000	-0.346088000
1	0.040083000	-4.525639000	-0.569313000	1	0.335920000	-4.454995000	-0.673757000
1	-0.647415000	-1.716740000	-1.293498000	1	-0.383444000	-1.614191000	-1.021419000
1	-0.649383000	-3.047616000	-2.257442000	1	-0.306199000	-2.763417000	-2.212711000
1	-1.799120000	-2.849462000	-1.055794000	1	-1.547739000	-2.790713000	-1.087205000
6	-0.332845000	-3.213666000	1.084499000	6	-0.138925000	-3.324429000	1.105212000
1	0.369197000	-3.741697000	1.733660000	1	0.460384000	-4.020496000	1.694773000
1	-0.275555000	-2.139420000	1.301274000	1	0.042979000	-2.315227000	1.493553000
8	-1.627749000	-3.704551000	1.275516000	8	-1.490262000	-3.677181000	1.194958000
1	-2.232827000	-2.938048000	1.300524000	1	-2.023442000	-2.862780000	1.183618000
6	1.595867000	-3.033742000	-0.532360000	6	1.805814000	-2.870746000	-0.439612000
8	1.818301000	-1.988107000	-1.229031000	8	1.912671000	-1.725916000	-0.994530000
8	2.432019000	-3.707981000	0.067406000	8	2.703967000	-3.536498000	0.069306000
1	-1.026592000	0.883966000	-0.958342000	1	-1.145092000	-0.340917000	0.639458000

The structures of L-serine clusters

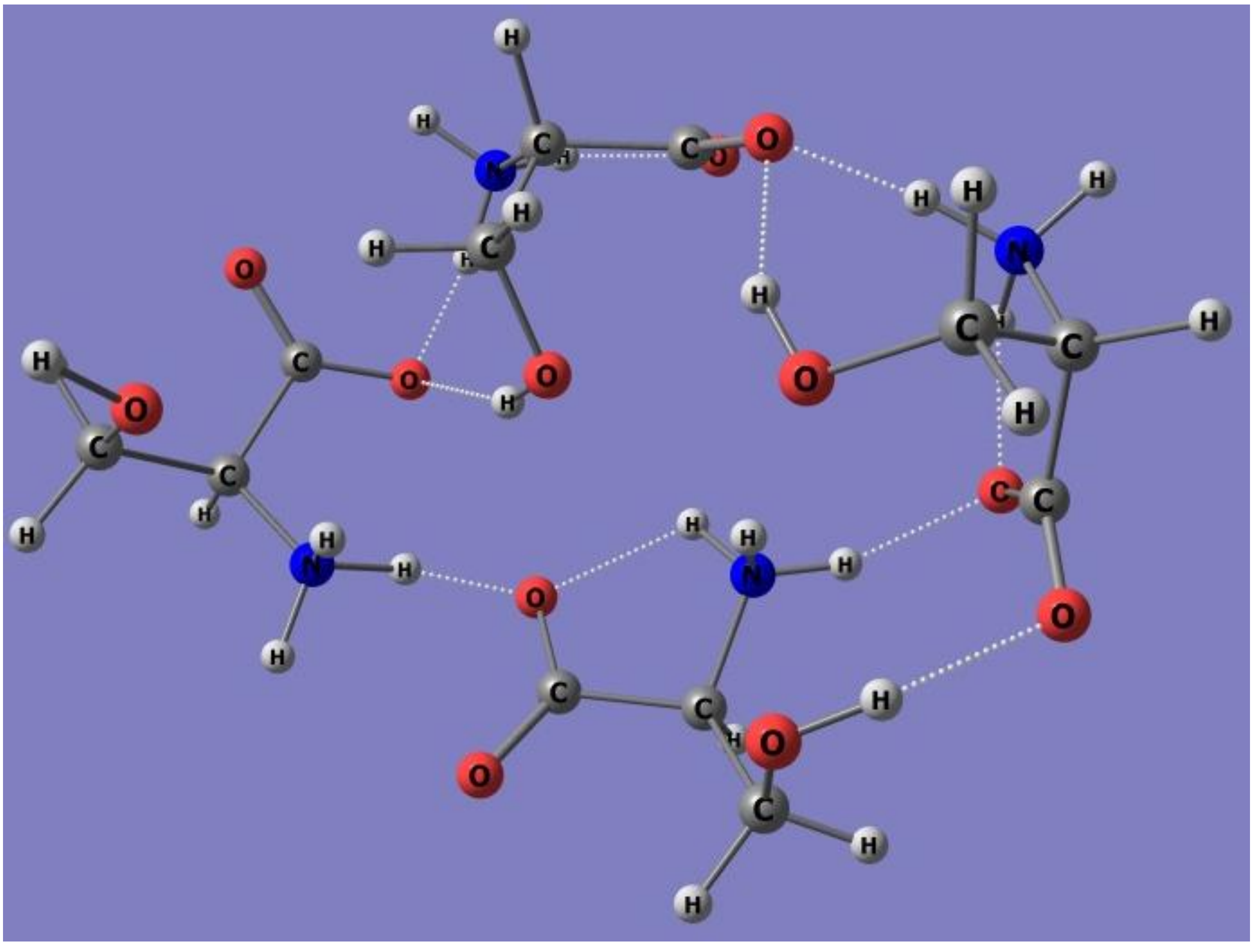

Figure S2. Structure of a cluster of four L-serine molecules, the DFT method with the ωb97xd basis set.

Table S7. Cartesian coordinates of atoms (XYZ, in angstroms) for a cluster of four L-serine molecules, DFT calculation method ωb97xd/6-311+G*.

Atom	XYZ, Å
N	-3.402862 1.743337 -0.648143
O	-1.433696 1.056771 1.566308
O	-0.756598 3.969750 0.339248
O	-1.161834 2.679229 -1.450709
C	-1.434760 3.163343 -0.339664
C	-2.754601 2.676884 0.315016
H	-3.409555 3.533773 0.484138
C	-2.472558 2.001265 1.656458
H	-2.154904 2.785622 2.346092
H	-3.382958 1.535632 2.046485
H	-2.679436 1.553026 -1.366377

H	-1.737222	0.132401	1.510985
H	-3.715377	0.816401	-0.215418
H	-4.193269	2.162663	-1.127180
N	2.732484	-0.939188	-0.491652
O	2.399366	-2.337713	1.893506
O	1.261945	-4.206054	-0.845824
O	0.434038	-2.128418	-0.702387
C	1.369760	-2.998096	-0.687685
C	2.775539	-2.415407	-0.428116
H	3.452208	-2.778085	-1.203553
C	3.294004	-2.856142	0.929387
H	3.318675	-3.950708	0.939041
H	4.310541	-2.470576	1.082464
H	2.004328	-0.538839	0.127086
H	2.726315	-2.522442	2.773356
H	3.617048	-0.447870	-0.237168
H	2.505038	-0.580759	-1.422323
N	1.585361	3.420968	-0.819365
O	1.152520	1.042733	0.773339
O	4.304642	1.211742	0.029416
O	3.062526	1.539189	-1.813207
C	3.431087	1.789929	-0.650297
C	2.589278	2.845096	0.116523
H	3.221186	3.647322	0.501186
C	1.869767	2.154596	1.272532
H	2.633023	1.823269	1.981187
H	1.190005	2.852221	1.771589
H	2.006878	4.100960	-1.445070
H	0.218358	1.095235	1.046728
H	1.245477	2.660396	-1.420836
H	0.728084	3.824594	-0.337123
N	-1.901118	-3.116142	-1.242144
O	-1.242299	-1.614850	1.402139
O	-3.894444	-0.645227	0.379495
O	-3.303759	-1.039733	-1.744621
C	-3.396784	-1.335770	-0.543538
C	-2.814452	-2.713811	-0.135060

H	-3.642318 -3.428540 -0.098780
C	-2.120261 -2.697896 1.224524
H	-2.896888 -2.600656 1.983212
H	-1.598830 -3.650440 1.387168
H	-0.896254 -2.726634 -1.074641
H	-0.462734 -1.723912 0.830064
H	-1.823048 -4.121343 -1.366619
H	-2.276792 -2.648843 -2.080666

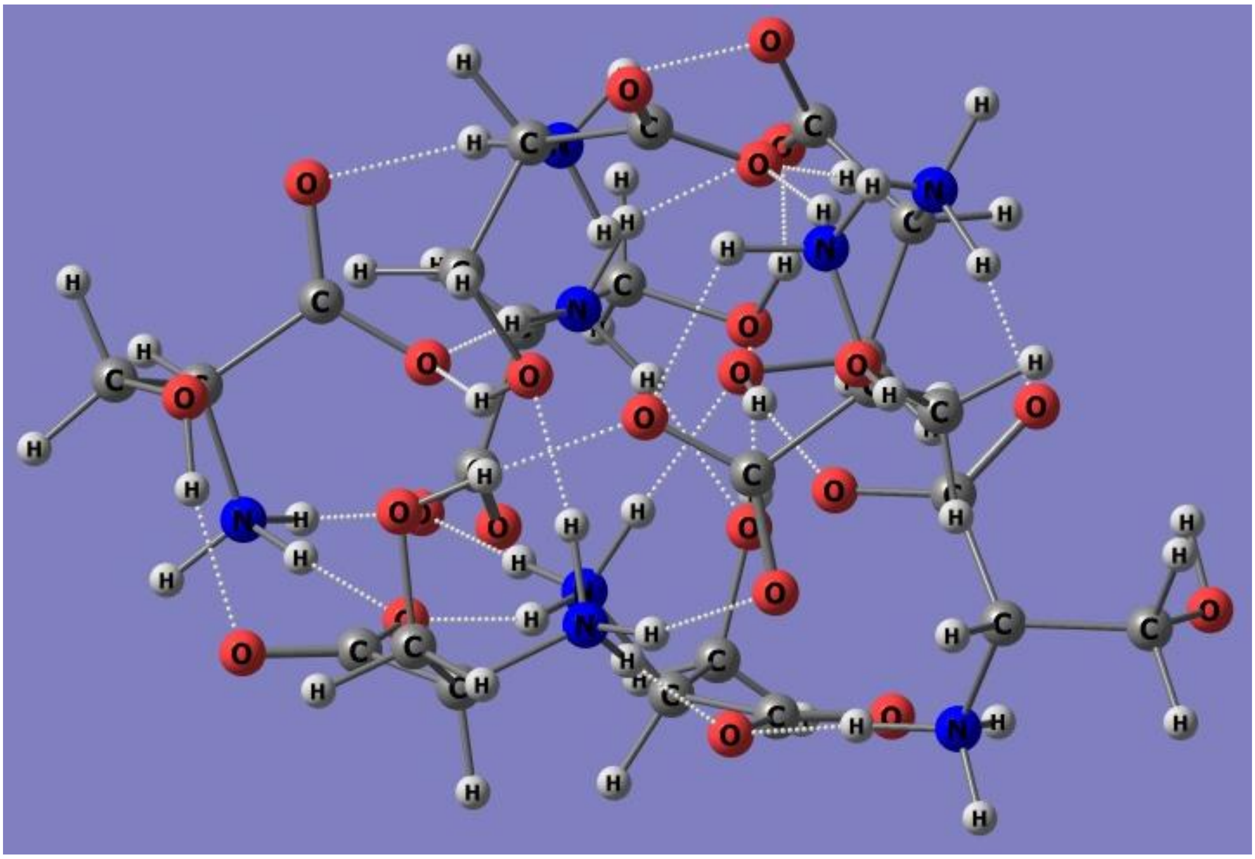

Figure S3. Structure of a cluster of eight L-serine molecules, the DFT method with the ωb97xd basis set.

Table S8. Cartesian coordinates of atoms (XYZ, in angstroms) for a cluster of eight L-serine molecules, DFT calculation method with the ωb97xd/6-31G* basis set.

Atom	XYZ, Å
H	-3.57998 -3.07468 1.91642
C	-4.27457 -2.703 1.16213
N	-4.50766 -1.25951 1.42793
H	-4.7579 -0.73958 0.56045

H	-3.65124 -0.77755 1.82408
C	-5.60827 -3.42846 1.26653
O	-6.18658 -3.06433 2.50953
C	-3.683 -2.88964 -0.25575
O	-2.86451 -3.81791 -0.39507
O	-4.13559 -2.09882 -1.12038
H	-5.26811 -1.16628 2.10717
H	-6.24532 -3.12304 0.42444
H	-5.4245 -4.50744 1.19754
H	-7.09804 -3.37541 2.53125
H	-3.68069 0.97737 -3.31903
C	-3.61902 1.12875 -2.23509
N	-2.7151 0.08096 -1.70404
H	-2.46523 0.2193 -0.71147
H	-1.80601 0.04865 -2.2008
C	-5.02204 0.98664 -1.65385
O	-5.0316 0.88349 -0.24686
C	-2.97198 2.5105 -1.99123
O	-3.69666 3.48633 -1.79995
O	-1.69645 2.48962 -2.02857
H	-3.16985 -0.85952 -1.71517
H	-5.60125 1.85259 -1.9914
H	-5.48739 0.07023 -2.02511
H	-4.49552 1.60764 0.1415
H	-1.06588 4.87025 1.71134
H	-3.11818 3.92887 2.23237
O	0.07303 2.07012 0.81057
C	-0.53053 2.81045 1.62794
O	-0.676 2.671 2.85956
C	-1.31145 4.00974 1.08703
H	-3.40113 4.22666 0.49243
C	-2.80529 3.66709 1.21707
N	-0.89469 4.34362 -0.29973
O	-3.078 2.29795 0.95923
H	-1.30565 5.22735 -0.60011
H	-2.73579 1.73334 1.68142
H	-1.21658 3.61099 -1.00321

H	0.86451 0.48435 1.09071
N	1.04537 -0.47976 1.43609
H	0.50044 1.76792 3.37429
H	1.98873 -0.53875 1.85723
O	1.17247 1.05551 3.56643
H	1.01538 -1.17194 0.68504
O	-2.29008 -0.08884 2.51538
C	-1.35187 -0.52269 1.79773
C	0.01827 -0.80665 2.4454
O	-1.44061 -0.83251 0.58168
C	0.41152 -0.11184 3.76436
H	1.03555 -0.80196 4.33928
H	0.05387 -1.89158 2.58454
H	-0.5034 0.097 4.32923
H	5.89405 -2.58479 1.54489
O	3.45669 -1.02699 2.67798
O	5.19284 0.01932 1.70192
C	4.48211 -0.97107 1.9521
H	5.59731 -1.48928 -0.56951
H	3.42316 -3.9281 0.98126
C	4.87606 -2.31229 1.25584
C	4.80714 -2.18383 -0.26342
H	4.98913 -3.15603 -0.73469
H	-0.0105 -4.97083 -1.21644
O	3.54276 -1.68814 -0.64041
H	0.65954 -3.97299 -3.45463
C	1.33085 -3.99264 -2.58704
H	1.91584 -4.91478 -2.62069
N	3.92603 -3.35392 1.7462
C	0.52288 -4.01803 -1.29155
H	3.19165 -2.8084 2.24025
H	3.13137 -2.22775 -1.35848
C	1.46242 -3.89768 -0.07029
O	2.50524 -4.5999 -0.10891
O	1.11976 -3.10918 0.8354
H	-1.47168 -3.3 -1.26919
H	4.35692 -3.98619 2.4178

N	-0.49147 -2.93274 -1.23111
H	-0.45273 -2.47996 -0.30046
O	2.21749 -2.89707 -2.62935
H	2.50039 1.7118 2.21775
H	1.89998 -2.19256 -3.24943
N	3.4057 2.19397 2.07535
H	3.20899 -0.04826 -0.47777
H	4.15065 1.4641 2.17903
H	-0.33556 -2.17376 -1.92807
O	2.81177 0.81422 -0.22007
H	3.43104 2.89157 2.82036
C	3.51751 2.88235 0.74982
O	1.89783 4.34462 1.64597
C	2.29686 3.83787 0.59258
H	-0.0746 1.71617 -1.46925
C	3.77122 1.85316 -0.33478
H	4.77272 1.43092 -0.21275
O	1.86704 4.04318 -0.58498
O	1.61725 -0.73646 -3.99574
H	4.40509 3.52212 0.81245
N	0.87954 1.79068 -1.84686
C	0.74713 -0.09748 -3.36119
H	3.70823 2.33933 -1.31206
O	-0.21583 -0.59269 -2.71932
C	2.17333 1.94268 -3.95335
C	0.8977 1.43831 -3.28674
H	1.5234 1.20678 -1.27647
H	1.2149 2.75145 -1.62418
H	2.07772 1.78987 -5.03676
H	0.01617 1.91276 -3.72764
H	0.15583 4.40763 -0.35783
O	3.32468 1.31765 -3.43972
H	2.28356 3.01683 -3.77169
H	3.20515 0.37971 -3.66724

Table S9. Results of calculations of L serine, n-Ser-L, clusters from n=1 to n=16 by the DFT B3LYP method.

n	E, a.u.	μ, D	HOMO, a.u.	LUMO, a.u.
1	-398.9501262	4.0747	-0.26015	0.00456
2	-797.9036289	2.3306	-0.24933	0.01283
3	-1196.9028102	3.8003	-0.23806	0.02139
4	-1595.8776943	6.8249	-0.22930	0.01658
6	-2393.8499006	11.3787	-0.20603	0.00044
8	-3191.8345023	7.5198	-0.23474	0.00357
10	-3989.7861934	8.3623	-0.23323	0.00443
12	-4787.74491920	6.7276	-0.23689	0.00228
14	-5585.67558046	6.1154	-0.24053	-0.00336
16	-6383.64478425	2.4652	-0.22601	-0.00089

Table S10. Results of calculations of L serine, n-Ser-L, clusters from n=1 to n=16 by the B3P86 DFT method.

n	E, au	μ, D	HOMO, eV	LUMO, eV
1	-399.9572578	4.1828	-0.28242	-0.01552
2	-799.9217813	2.2692	-0.27618	-0.00911
3	-1199.9276825	3.7002	-0.26320	0.00044
4	-1599.9067393	6.1654	-0.25609	0.00030
6	-2399.8963472	9.3869	-0.23709	-0.01744
8	-3199.8972499	7.6241	-0.25915	-0.01988
10	-3999.8450394	7.8897	-0.25747	-0.00100
12	-4799.8221968	4.1929	-0.25835	-0.02605
14	-5599.7746392	5.7541	-0.26372	-0.02112
16	-6399.7459244	4.4745	-0.25009	-0.02227

Table S11. Results of calculations of L serine, n-Ser-L, clusters from n=1 to n=16 by the DFT M06 method.

**n**	***E*, au**	**μ, D**	**HOMO, eV**	**LUMO, eV**
1	-398.7409062	4.0784	-0.27487	0.01462
2	-797.4877755	2.6339	-0.26214	0.01907

3	-1196.2848045	3.2062	-0.25081	0.03591
4	-1595.0439223	6.3463	-0.24190	0.03197
6	-2392.6230892	7.3818	-0.23610	0.01557
8	-3190.2135254	7.3666	-0.24706	0.01485
10	-3987.7362141	7.0070	-0.24539	0.00737
12	-4785.2910229	5.1240	-0.25065	0.00667
14	-5582.8393165	4.5820	-0.24873	0.00569
16	-6380.3355674	5.4941	-0.24538	0.00588

Table S12. Calculated bond lengths and bond angles for the complexes **1**–**8**.

Complex	R(X-H), Å	R(XH…Y),* Å	□(X-H…Y), deg.
L-Ser-M-H_2O_2 (**1**)	0.981<br>0.980<br>0.993	1.956<br>1.837<br>1.852	145.28<br>157.54<br>127.57
D-Ser-M-H_2O_2 (**2**)	0.982<br>0.976<br>0.991	1.954<br>2.242<br>1.899	163.19<br>146.97<br>126.72
L-Ser-P-H_2O_2 (**3**)	0.975<br>0.984<br>0.995	1.947<br>1.816<br>1.840	160.57<br>158.17<br>128.06
D-Ser-P-H_2O_2 (**4**)	0.986<br>0.996	1.782<br>1.839	169.82<br>128.32
-Ser-M-H_2O_2/ H_2O (**5**)	0.989<br>0.992	1.777<br>1.730	170.74<br>169.01
D-Ser-M-H_2O_2/ H_2O (**6**)	1.001<br>0.975	1.694<br>2.565	174.74<br>149.97
L-Ser-P-H_2O_2/ H_2O (**7**)	0.980<br>0.998	1.885<br>1.681	169.29<br>173.10
D-Ser-P-H_2O_2/ H_2O (**8**)	1.000	1.646	176.53

***)** R(XH…Y) – hydrogen bond

CD spectra of the studied complexes

Figures S4-S7 show the calculated CD spectra of the studied complexes obtained using four different DFT methods.

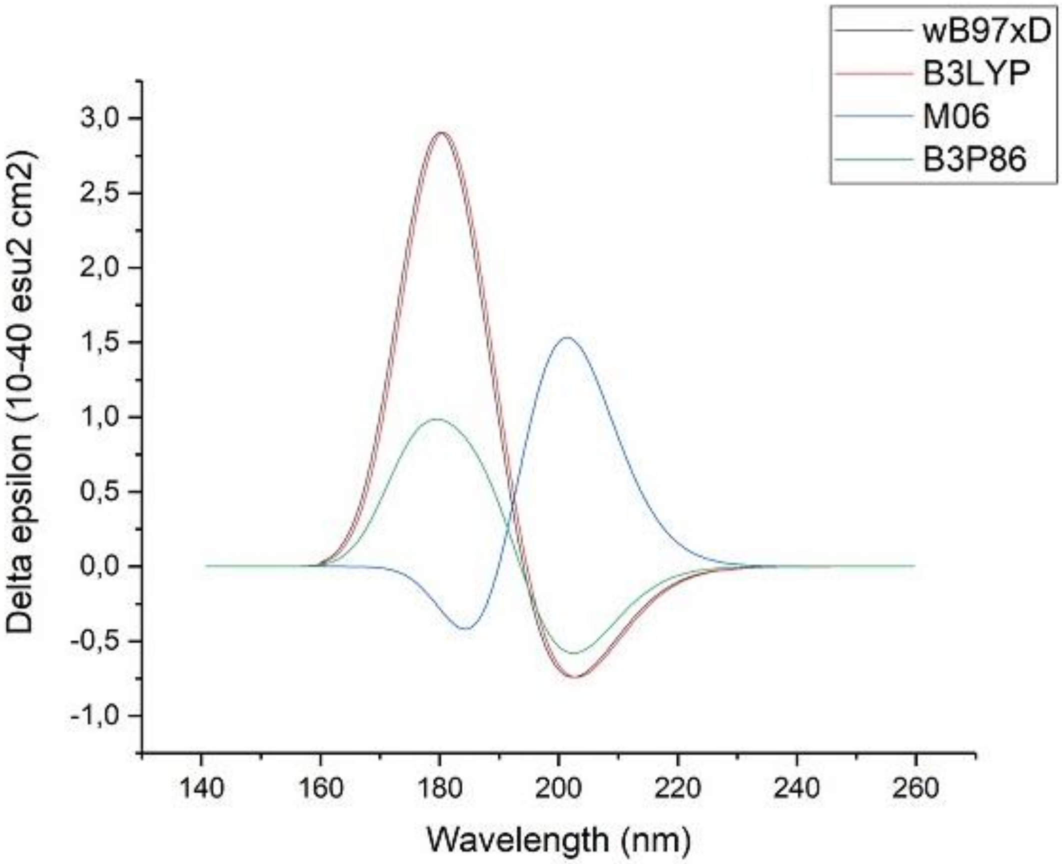

Figure S4. Circular dichroism spectra of complexes of two molecules of hydrogen peroxide in M form with clusters of two molecules of D-enantiomers of serine in aqueous solution obtained using different DFT methods.

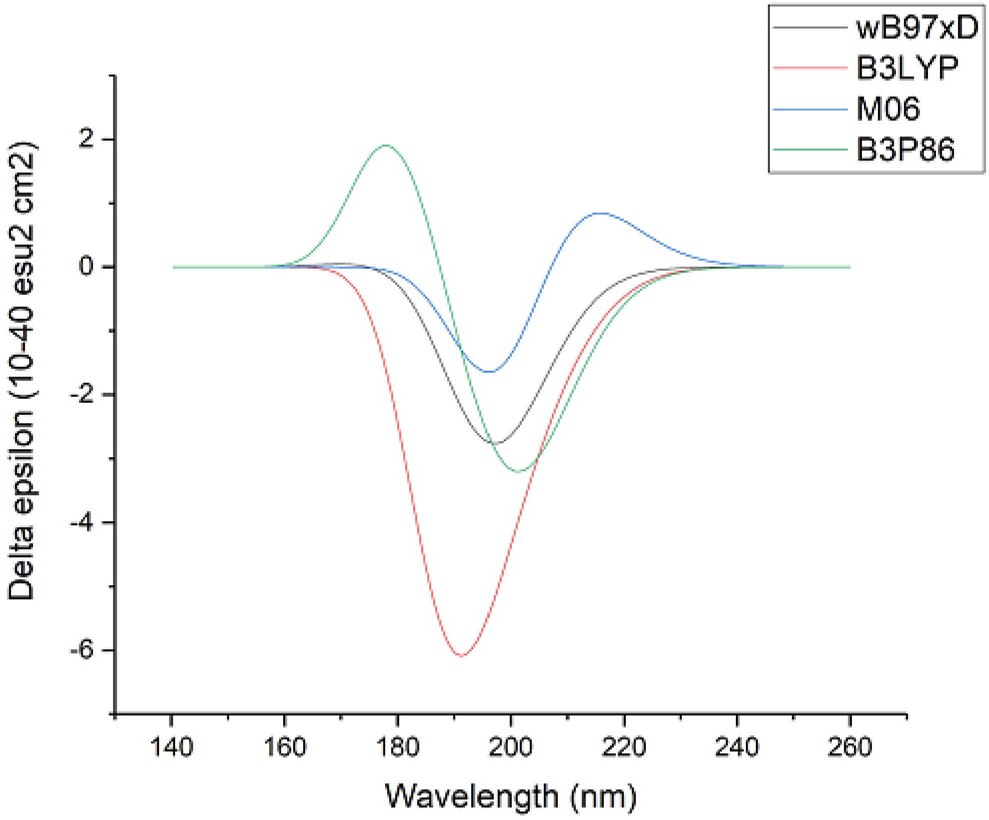

Figure S5. Circular dichroism spectra of clusters of two molecules of L-enantiomers of serine in aqueous solution obtained using different DFT methods.

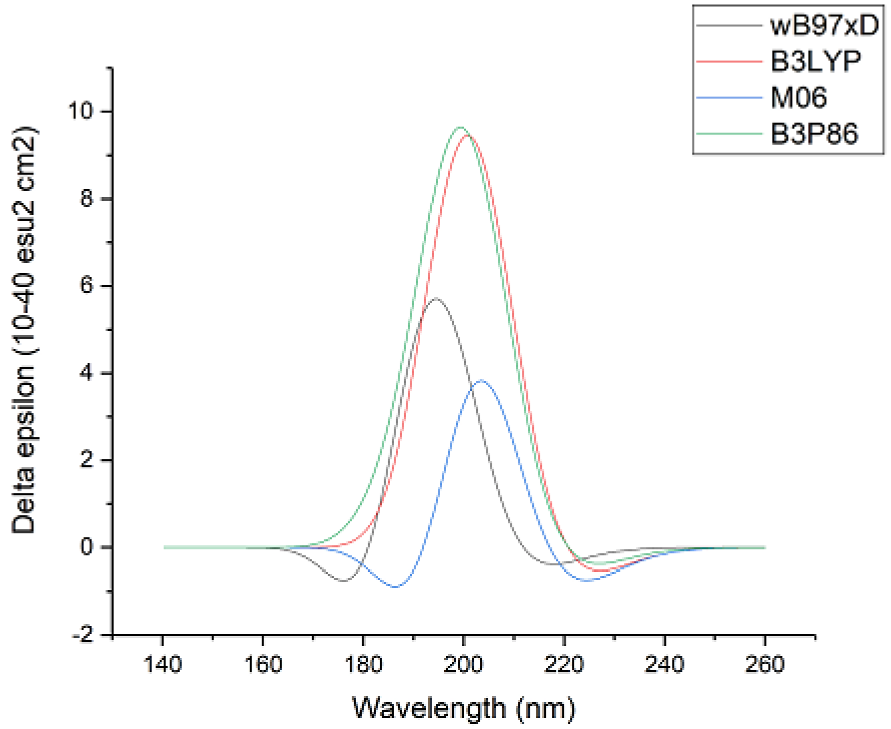

Figure S6. Circular dichroism spectra of complexes of two molecules of hydrogen peroxide in M form with clusters of two molecules of L-enantiomers of serine in aqueous solution obtained using different DFT methods.

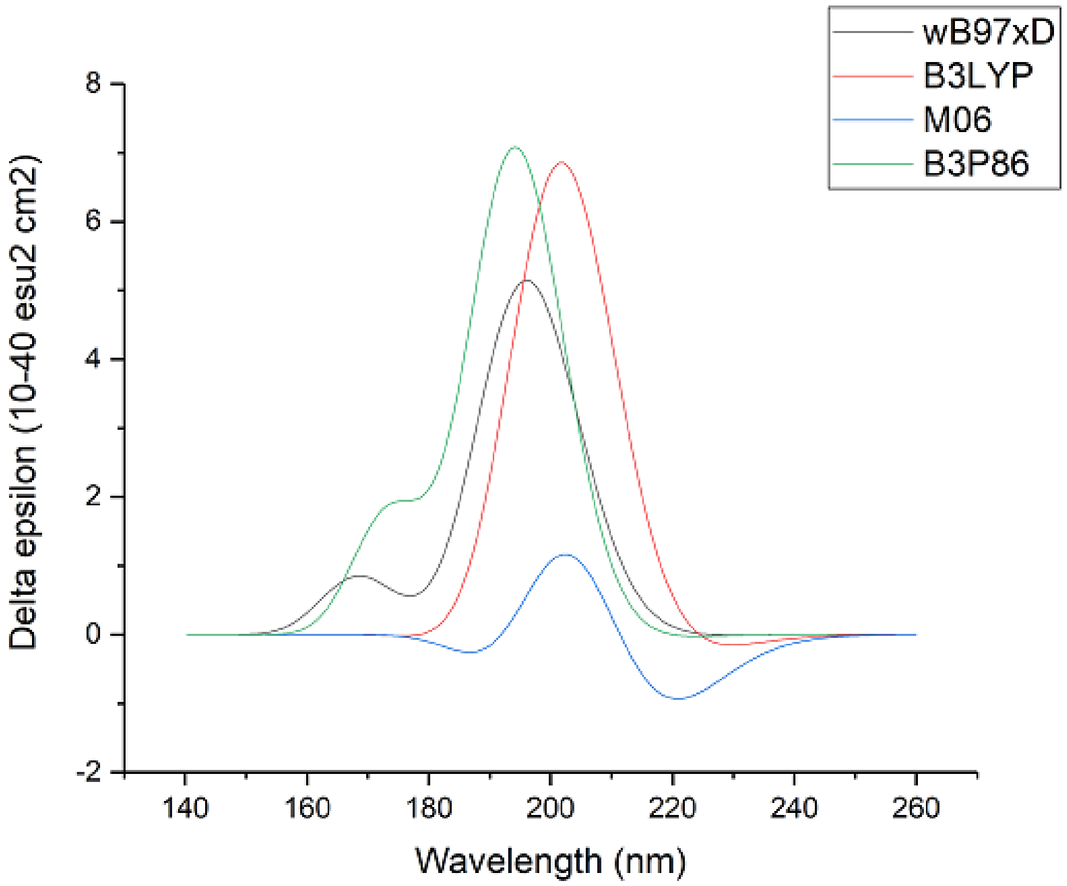

Figure S7. Circular dichroism spectra of clusters of two molecules of D-enantiomers of serine in aqueous solution obtained using different DFT methods.

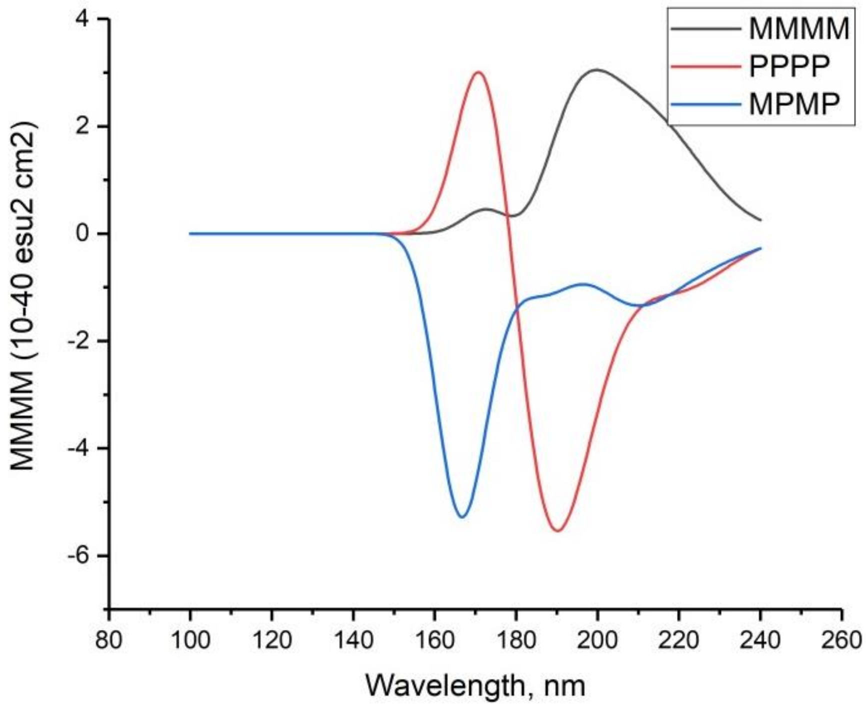

Figure S8. Calculated CD spectra of hydrogen peroxide in M and P forms and its tetrameric clusters; MP2/6-311+G**.
